# The mechanism of Annexin A1 to modulate TRPV1 and nociception in dorsal root ganglion neurons

**DOI:** 10.1186/s13578-021-00679-1

**Published:** 2021-08-26

**Authors:** Yufen Zhang, Sehui Ma, Xiao Ke, Yao Yi, Hongyan Yu, Dian Yu, Qiang Li, You Shang, Youming Lu, Lei Pei

**Affiliations:** 1 Department of Neurobiology, School of Basic Medicine, Tongji Medical College, Huazhong University of Science and Technology, Wuhan, 430030 China; 2 Collaborative Innovation Center for Brain Science, The Institute for Brain Research (IBR), Huazhong University of Science and Technology, Wuhan, 430030 China; 3 Exchange, Development & Service Center for Science & Technology Talents, The Ministry of Science and Technology (Most), Beijing, 100045 China; 4 Department of Critical Care Medicine, Union Hospital, Tongji Medical College, Huazhong University of Science and Technology, Wuhan, 430022 China; 5 Department of Physiology, School of Basic Medicine, Tongji Medical College, Huazhong University of Science and Technology, Wuhan, 430030 China; 6 Department of Anesthesiology, School of Medicine, Washington University in Saint Loius, St. Loius, MO 63110 USA

**Keywords:** Annexin A1, Formyl peptide receptor, Neuronal sensitivity, Nociceptive sensation, Transient receptor potential vanilloid 1

## Abstract

**Background:**

Annexin A1 (ANXA1) exerts anti-nociceptive effect through ANXA1 receptor formyl peptide receptor 2 (FPR2/ALX (receptor for lipoxin A4), FPR2) at the dorsal root ganglia (DRG) level. However, the mechanisms remain elucidated. By using radiant heat, hot/cold plate, tail flick, von Frey, and Randall-Selitto tests to detect nociception in intact and chemical (capsaicin, menthol, mustard oil, formalin or CFA) injected *AnxA1* conditional knockout (*AnxA1*^−/−^) mice, applying calcium imaging and patch clamp recordings in cultured DRG neurons to measure neuronal excitability, conducting immunofluorescence and western blotting to detect the protein levels of TRPV1, FPR2 and its downstream molecules, and performing double immunofluorescence and co-immunoprecipitation to investigate the interaction between Calmodulin (CaM) and TRPV1; we aim to uncover the molecular and cellular mechanisms of ANXA1’s role in antinociception.

**Results:**

*AnxA1*^−/−^ mice exhibited significant sensitivity to noxious heat (mean ± SD, 6.2 ± 1.0 s *vs*. 9.9 ± 1.6 s in Hargreaves test; 13.6 ± 1.5 s *vs*. 19.0 ± 1.9 s in hot plate test; n = 8; *P* < 0.001), capsaicin (101.0 ± 15.3 *vs*. 76.2 ± 10.9; n = 8; *P* < 0.01), formalin (early phase: 169.5 ± 32.8 s *vs*. 76.0 ± 21.9 s; n = 8; *P* < 0.05; late phase: 444.6 ± 40.1 s *vs*. 320.4 ± 33.6 s; n = 8; *P* < 0.01) and CFA (3.5 ± 0.8 s *vs*. 5.9 ± 1.4 s; n = 8; *P* < 0.01). In addition, we found significantly increased capsaicin induced Ca^2+^ response, TRPV1 currents and neuronal firing in *AnxA1* deficient DRG neurons. Furthermore, ANXA1 mimic peptide Ac2-26 robustly increased intracellular Ca^2+^, inhibited TRPV1 current, activated PLCβ and promoted CaM-TRPV1 interaction. And these effects of Ac2-26 could be attenuated by FPR2 antagonist Boc2.

**Conclusions:**

Selective deletion of *AnxA1* in DRG neurons enhances TRPV1 sensitivity and deteriorates noxious heat or capsaicin induced nociception, while ANXA1 mimic peptide Ac2-26 desensitizes TRPV1 via FPR2 and the downstream PLCβ-Ca^2+^-CaM signal. This study may provide possible target for developing new analgesic drugs in inflammatory pain.

**Supplementary Information:**

The online version contains supplementary material available at 10.1186/s13578-021-00679-1.

## Introduction

Though currently available analgesics including opioids and cannabinoids have therapeutic benefits, long-term use causes unintended toxicity and serious side effects [1]. Nonsteroidal medications are potentially harmful to the heart, liver, and kidney when used as adjunctive analgesics [2]. Therefore, it is necessary to develop novel analgesics with low toxicity and side effects.

Annexin A1 (ANXA1) is a calcium and phospholipid binding protein that belongs to the annexin superfamily. Studies show ANXA1 effectively alleviates nociception elicited by a variety of nociceptive stimuli [3, 4]. Our previous study in rats found that intrathecal injecting ANXA1 mimic peptide Ac2-26 into the lumbar region effectively decreased hyperalgesia in complete Freund's adjuvant (CFA) induced inflammatory pain [5]. Furthermore, intrathecal injection of BMS-986235, an agonist of ANXA1 receptor formyl peptide receptor 2 (FPR2) mimicked the anti-nociceptive effects of Ac2-26, which can be reverted by the FPR2 blocker Boc2 [6–8]. Both ANXA1 and FPR2 were found to be strongly expressed in dorsal root ganglion (DRG) neurons and spinal dorsal horn, which are linked to the development of nociception [5]. Recently, Christabel Fung et al. reported that the spinal cord contained high levels of FPR2 protein [9]. These studies indicate that ANXA1 may elicit anti-nociceptive effect through FPR2 both peripherally and centrally.

TRPV1 is considered to be a detector and integrator of nociception, and modulation of TRPV1 is implicated in hypersensitivity, and shown to contribute to several conditions of pain [10]. Although numerous studies have demonstrated the role of TRPV1 in pain, whether ANXA1-FPR2 regulates the sensitivity of TRPV1 to participate in nociception is unclear.

In this study, by using conditional knockout mice, combining a variety of pain behavioral measurements, Ca^2+^ imaging, whole cell patch recording and co-immunoprecipitation, we investigated whether deletion of ANXA1 in DRG affects nociception and TRPV1 function through FPR2 signal in DRG neurons. We aim to elucidate the mechanism of ANXA1 to modulate TRPV1-mediated nociception in mice.

## Results

### ***AnxA1***^***−/−***^ mice are more sensitive to noxious heat stimuli and capsaicin induced nociception

**Fig. 1:**
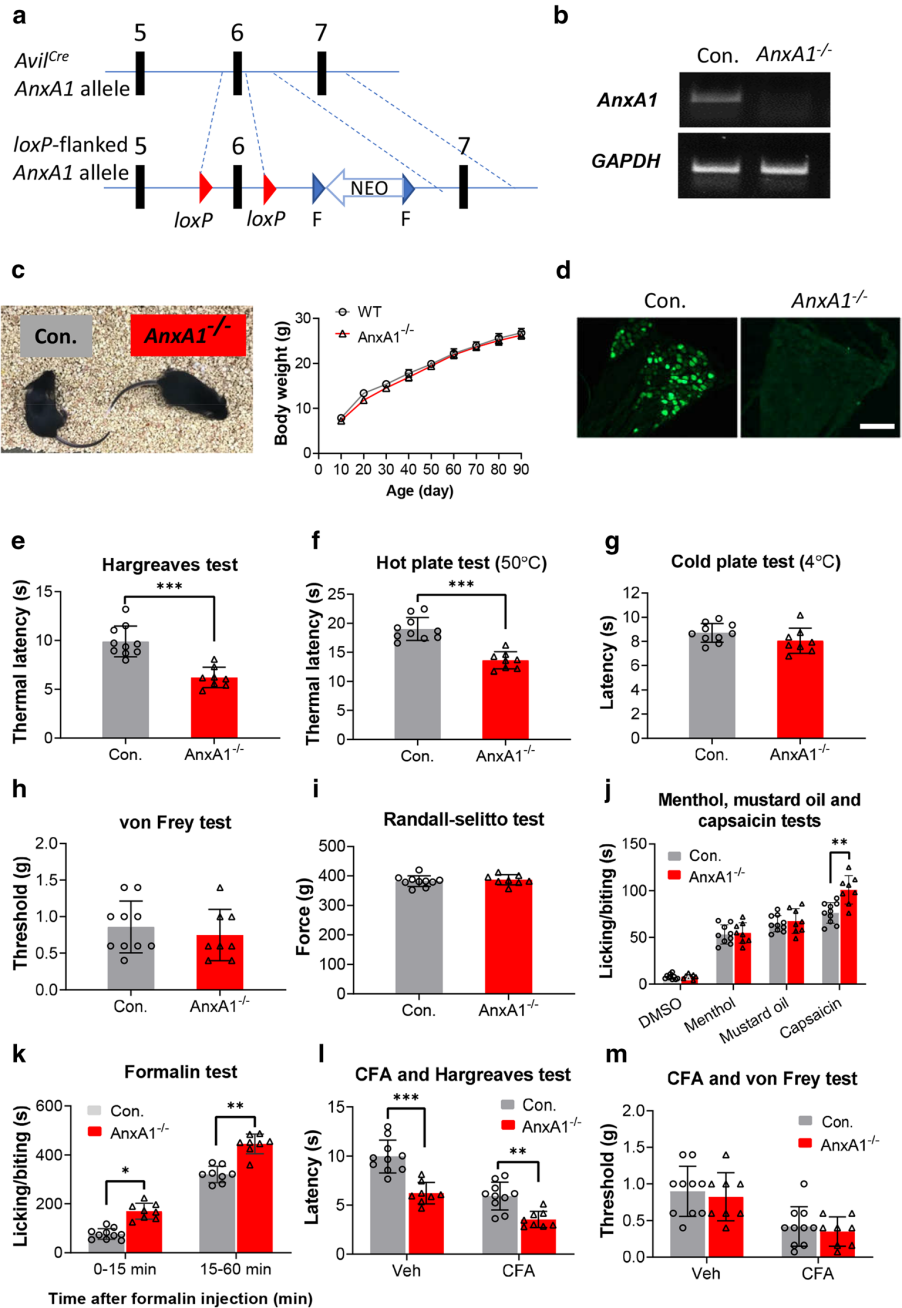
Generating of *AnxA1*^−/−^ mice and detecting nociceptive sensation. **a** Gene targeting strategy. Two *loxP* sites were inserted on both sides of exon 6 of the AnxA1 locus followed by a FRT-flanked NEO cassette (F and NEO). To selectively delete *AnxA1* gene in DRG neurons, we crossed *AnxA1*^*loxP/*+^ mice with *Avil*^*cre*^ mice to eventually obtain *Avil*^*cre*^-*AnxA1*^*loxP/loxP*^ (*AnxA1*^*−/−*^) mice and littermate controls (*Avil*^*cre*^-*AnxA1*^+/+^, Con.). **b** DNA band image of *AnxA1* in control and *AnxA1*^*−/−*^ mice from PCR genotyping results. **c** The gross physical appearance and the body weight gain in control and *AnxA1*^*−/−*^ mice. **d** Immunofluorescent staining of DRG frozen sections isolated from control and *AnxA1*^*−/−*^ mice stained with antibody to ANXA1. Scale bar, 100 µm. Measurement of thermal (**e**–**g**) and mechanical (**h** and **i**) nociception between control and *AnxA1*^*−/−*^ mice. **e** Quantification of the thermal latency to radiant heat. **f** Quantification of the thermal latency to hot plate at 50 ℃ (*AnxA1*^*−/−*^ versus control group, ***P < 0.001, Student’s t-test). **g** Quantification of the response latency to cold plate at 4 ℃. **h** Quantification of the threshold to von Frey filaments. **i** Quantitative analysis of mechanical pressure force in Randall-Selitto test (*AnxA1*^*−/−*^ versus control group, P > 0.05, Student’s t-test). Measurement of chemical nociception (j and k) between control and *AnxA1*^*−/−*^ mice. **j** Quantitative analysis of the licking or biting duration over 10 min after unilateral injection of 40 μg menthol, 5% mustard oil or 25 μg capsaicin into the hindpaw of mice (*AnxA1*^*−/−*^ versus control, **P < 0.01, Student’s t test). **k** Quantitative analysis of the licking or biting duration over 60 min after injection of 1% formalin into the hindpaw of mice (*AnxA1*^*−/−*^ versus control, *P < 0.05, **P < 0.01, Student’s t-test). Quantitative analysis of the withdrawal latency to radiant heat in Hargreaves test (**l**) and threshold in von Frey test (**m**) after unilateral injection of CFA into the hindpaw of mice (*AnxA1*^*−/−*^ versus control, **P < 0.01, ***P < 0.001, two-way ANOVA followed by Sidak’s multiple comparisons test, n = 10 in control group, n = 8 in *AnxA1*^*−/−*^ group). All data are represented as mean ± SD

We previously demonstrated that ANXA1 has anti-nociceptive effects through FPR2 at the DRG level [5]. Therefore, we asked whether selective deletion of ANXA1 in DRG affects nociception. We generated *AnxA1* conditional knockout (*AnxA1*^*−/−*^) mice (Fig. 1a) and explored the consequences of *AnxA1*^*−/−*^ in thermal, mechanical, chemical and inflammatory nociception. The control littermates (Con.), and homozygous (*AnxA1*^*−/−*^) knockout mice were validated by genotyping (Fig. 1b). *AnxA1*^*−/−*^ mice showed normal hair, body shape and body weight as compared with control littermates (Fig. 1 c). Immunofluorescence staining confirmed that ANXA1 was completely abolished in the DRG (Fig. 1d).

Pain behavioral results showed that the thermal latency of *AnxA1*^*−/−*^ mice was significantly decreased in both Hargreaves test (6.2 ± 1.0 s) and hot plate test (13.6 ± 1.5 s) as compared with control littermates (9.9 ± 1.6 and 19.0 ± 9.9 s, respectively) (*P* < 0.001; Fig. 1e, f). However, no difference was found in cold plate assay (Fig. 1g) test, von Frey test (Fig. 1h), or Randall-Selitto test (Fig. 1i) between *AnxA1*^*−/−*^ and control mice. Among chemical stimuli, only intraplantar injection of capsaicin (25 µg), but not menthol (40 µg) or mustard oil (5%), induced exacerbated licking/biting behavior in *AnxA1*^*−/−*^ mice (101.0 ± 15.3 times) compared with control (76.2 ± 10.9 times) (*P* < 0.01; Fig. 1j). These results suggest that *AnxA1*^*−/−*^ mice exhibit increased responsiveness to noxious thermal stimuli and capsaicin-induced nociception.

In the formalin test, the licking/biting time was significantly increased both in the early phase (0–15 min, 169.5 ± 32.7 s *vs.* 76.0 ± 21.9 s) and later phase (15–60 min, 444.6 ± 40.1 s *vs.* 320.4 ± 33.6 s) after injection of formalin into one hindpaw of *AnxA1*^*−/−*^ mice (*P* < 0.05 in 0–15 min; *P* < 0.01 in 15–60 min; Fig. 1k). Our previous study showed that the pain response peaked one week after the CFA injection. Therefore, in this study, the Hargreaves test and von Frey test were performed on day 7 after unilateral intraplantar injection of CFA. We observed that *AnxA1*^*−/−*^ mice displayed a significant reduction in thermal latency (Veh: 6.2 ± 1.1 s, CFA: 3.6 ± 0.8 s) compared to control littermates (Veh: 9.9 ± 1.7 s, CFA: 5.9 ± 1.4 s) (*P* < 0.01; Fig. 1l). However, the threshold of von Frey test has no obvious difference between *AnxA1*^*−/−*^ and control mice in either Veh or CFA group (*P* = 0.83; *P* = 0.85; Fig. 1m). These results suggest that *AnxA1*^*−/−*^ mice exhibit increased spontaneous pain and inflammatory thermal hyperalgesia.

### *AnxA1* deficiency does not affect the expression of FPR2 and nociceptive receptors in DRG neurons

**Fig. 2:**
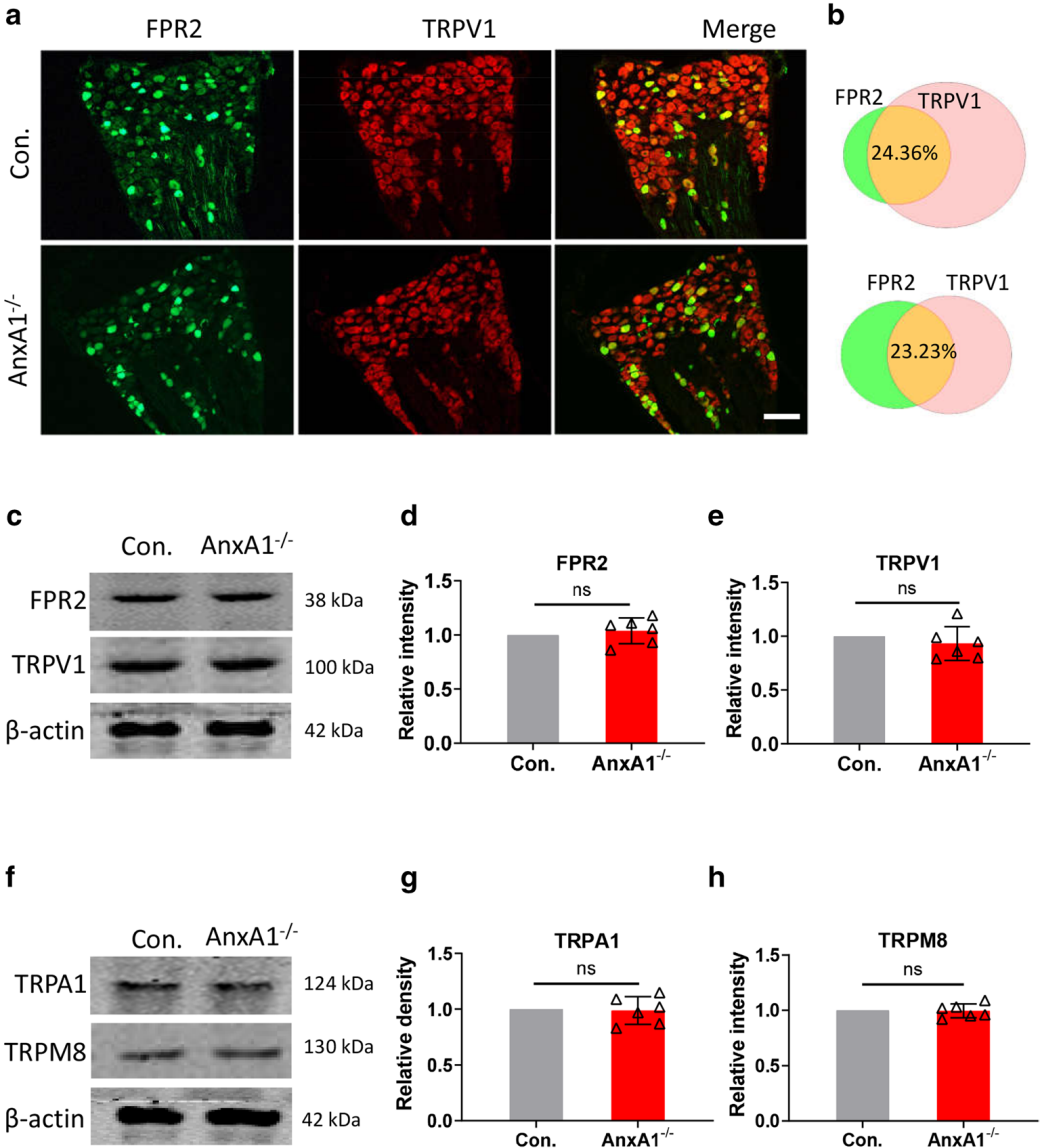
*AnxA1* deletion does not alter the expression of FPR2, TRPV1 and other nociceptors. **a** Representative images of double immunofluorescent staining and **b** Venn diagram of the percentages of FPR2 positive and TRPV1 positive neurons in DRG sections from *AnxA1*^*−/−*^ mice and WT littermates labeled for FPR2 and TRPV1 as indicated. Scale bar, 100 μm. **c** Representative western blots band images and the quantification of the indicated proteins **d** ANXA1 (*AnxA1*^*−/−*^ versus control group, P = 0.45, Student’s t-test. n = 6 in control group, n = 6 in *AnxA1*^*−/−*^ group), and **e** TRPV1 *AnxA1*^*−/−*^ versus control group, *P* = 0.32, Student’s t-test. n = 6 in WT group, n = 6 in *AnxA1*^*−/−*^ group) in DRG tissues between *AnxA1*^*−/−*^ mice and control littermates. **f** Representative band images and the quantification of other nociceptors such as **g** TRPA1 and **h** TRPM8 in L4-6 DRG tissues between *AnxA1*^*−/−*^ mice and control littermates (*AnxA1*^*−/−*^ versus control, P = 0.82, P = 0.85, Student’s t-test. n = 6 in control group, n = 6 in *AnxA1*^*−/−*^ group). The relative density of the protein band image in control group was normalized to 1. All data are represented as mean ± SD

Since nociceptive responses induced by noxious heat and capsaicin were enhanced in *AnxA1*^*−/−*^ mice, and TRPV1 has no binding site of ANXA1 [5], we speculate that *AnxA1* deficiency in DRG might affect the expression of FPR2 or TRPV1. We detected the co-localizations of TRPV1 with FPR2 in mice L4-6 DRGs neurons by using double immunofluorescent staining. Both FPR2 and ANXA1 were found to be dispersed and colocalized with TRPV1 in the DRG neurons of control mice (Fig. 2a, Additional file 1a). *AnxA1* deletion has no effect on FPR2 and TRVP1 expression in DRG neurons (Fig. 2a). The average number of FPR2 positive (FPR2^+^) neurons is 49.0 ± 10.4 and 50.7 ± 9.9 in control and *AnxA1*^*−/−*^ mice, respectively. And the average number of TRPV1 positive (TRPV1^+^) neurons is 128.4 ± 11.4 and 122.8 ± 11.1 in control littermates and *AnxA1*^*−/−*^ mice, respectively (*P* = 0.45; *P* = 0.78; Additional file 1b). In addition, the average percentage of FPR2 and TRPV1 double positive (FPR2^+^/TRPV1^+^) neurons are highly close in the two genotypes (24.36 and 23.23%, respectively) (Fig. 2b).

Next, we further analyzed the protein levels of ANXA1, FPR2 and TRPV1 in L4-6 DRGs by western blotting. Consistent with the staining results, ANXA1 protein was absent in the *AnxA1*^*−/−*^ mice (Additional file 1c), but abundantly expressed in the DRGs of the control littermates without significantly affecting the expression of FPR2 (*P* = 0.45; Fig. 2c, d). Furthermore, the protein levels of TRPV1, TRPA1 and TRPM8 in the DRGs also have no difference between *AnxA1*^*−/−*^ and control mice (*P* = 0.32; *P* = 0.82; *P* = 0.85; Fig. 2c, e–h). In addition, FPR2 showed similar co-labeling pattern with TRPA1, and TRPM8 (the overlapping rates are 12.3, and 16.6% respectively) (Additional file 2). Thus, *AnxA1* deletion has no effect on FPR2 expression or FPR2 co-localization with TRPV1 in DRG neurons.

### *AnxA1* deficiency increases capsaicin induced Ca^2+^ responses in DRG neurons

**Fig. 3:**
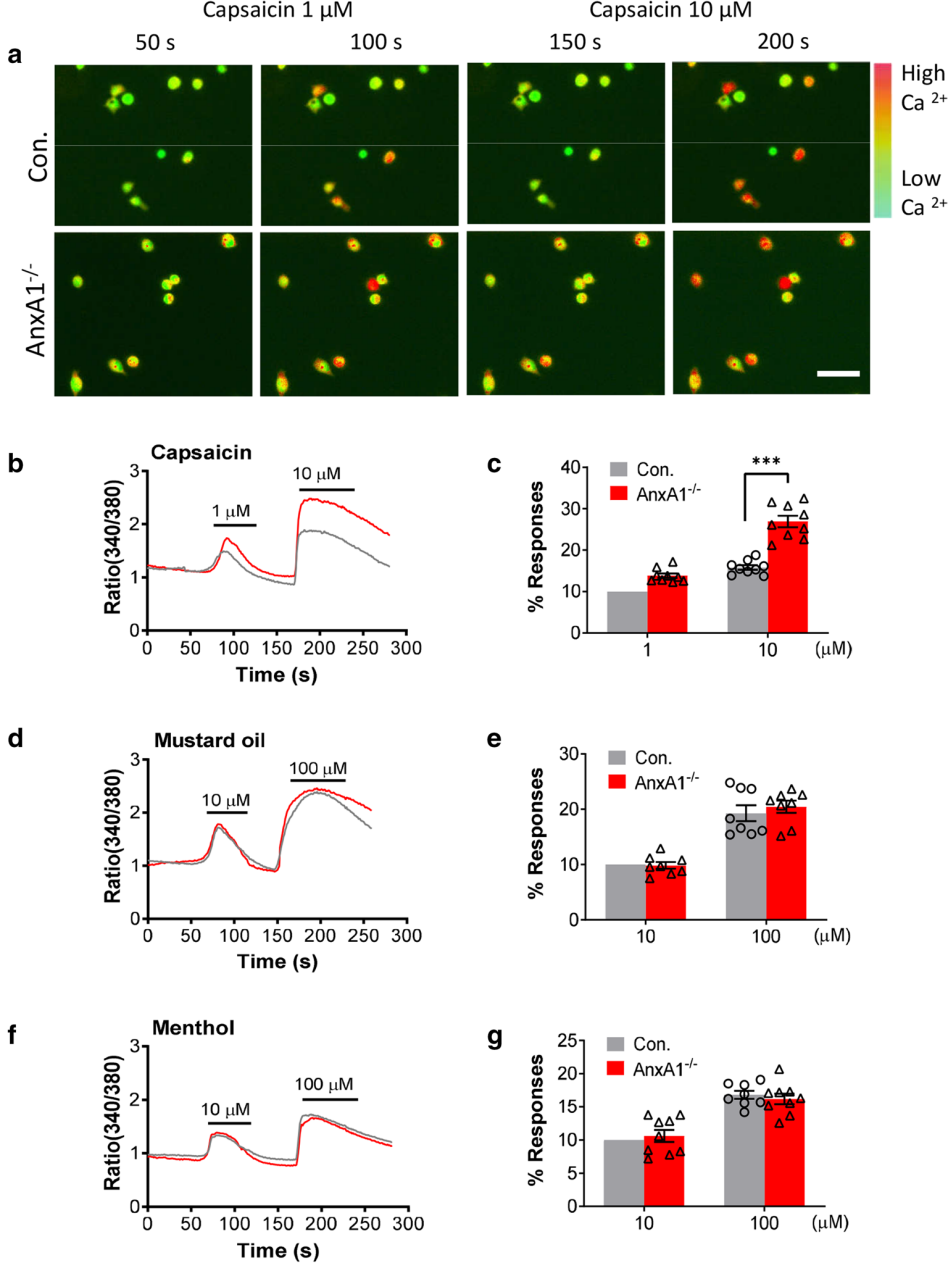
*AnxA1* deletion potentiates capsaicin induced calcium responses in DRG neurons. **a** Representative images of the calcium-dependent fluorescence before and after application with capsaicin (1 µM and 10 µM) in cultured DRG neurons between control mice (upper panels) and *AnxA1*^*−/−*^ mice (lower panels). Green color indicates basal intracellular Ca^2+^ concentration (i.e. before depolarization) in Fura-2 dye loaded DRG neurons. Warmer colors (yellow, orange and red) indicate that the cytoplasmic Ca^2+^ concentration is relatively higher. Scale bar, 100 μm. Representative traces showing the time course of changes in ratio (340/380) of calcium-dependent fluorescence during application of **b** and **c** capsaicin (1 µM and 10 µM), **d** and **e** mustard oil (10 µM  and 100 µM), and **f** and **g** menthol (10 µM and 100 µM). Quantified data showing the percentage of calcium responses after application of **c** capsaicin (1 µM and 10 µM, *AnxA1*^*−/−*^ versus control group, ***P < 0.001, Student’s t-test. n = 9 in control group, n = 9 in *AnxA1*^*−/−*^ group), **e** mustard oil (10 µM and 100 µM, *AnxA1*^*−/−*^ versus control group, P = 0.99, P = 0.64, Student’s t-test. n = 8 in control group, n = 8 in *AnxA1*^*−/−*^ group), and **g** menthol (10 µM and 100 µM, *AnxA1*^*−/−*^ versus control group, P = 0.78, P = 0.78, Student’s t-test. n = 8 in control group, n = 9 in *AnxA1*^*−/−*^ group) in DRG neurons between *AnxA1*^*−/−*^ mice and control littermates. Responses percentage in control group was normalized to 10%. All data are represented as mean ± SD

Since *AnxA1* deletion has no effect on TRPV1 expression, we suppose the increased nociceptive response after *AnxA1* deletion is due to increased sensitivity of DRG neurons to nociceptive stimuli. To verify this hypothesis, we detected capsaicin mediated Ca^2+^ response by using ratiometric calcium imaging in cultured neurons derived from the L4-6 DRGs of *AnxA1*^−/−^ mice and control littermates. Our results showed that 1 µM capsaicin induced a slight increase of Ca^2+^ response during 30 s applications, which was recovered toward baseline following 50 s. Whereas 10 µM capsaicin elicited a robust increase of Ca^2+^ response in cultured DRG neurons of *AnxA1*^−/−^ mice compared to cultures of control mice (20.4 ± 9.2 *vs*. 12.9 ± 4.1%, n = 9, *P* < 0.001; Fig. 3a–c). However, we did not observe any difference of Ca^2+^ response between the two genotype groups when DRG neurons treated with mustard oil (10, 100 µM) (*P* = 0.99; *P* = 0.64; Fig. 3d, e), or menthol (10, 100 µM) (*P* = 0.78; *P* = 0.78; Fig. 3f, g) either at lower or higher concentration. Taken together, the results indicate that ANXA1 specifically modulates TRPV1 responses, but not TRPA1 or TRPM8.

### *AnxA1* deficiency enhances capsaicin induced TRPV1 sensitivity in DRG neurons.

**Fig. 4:**
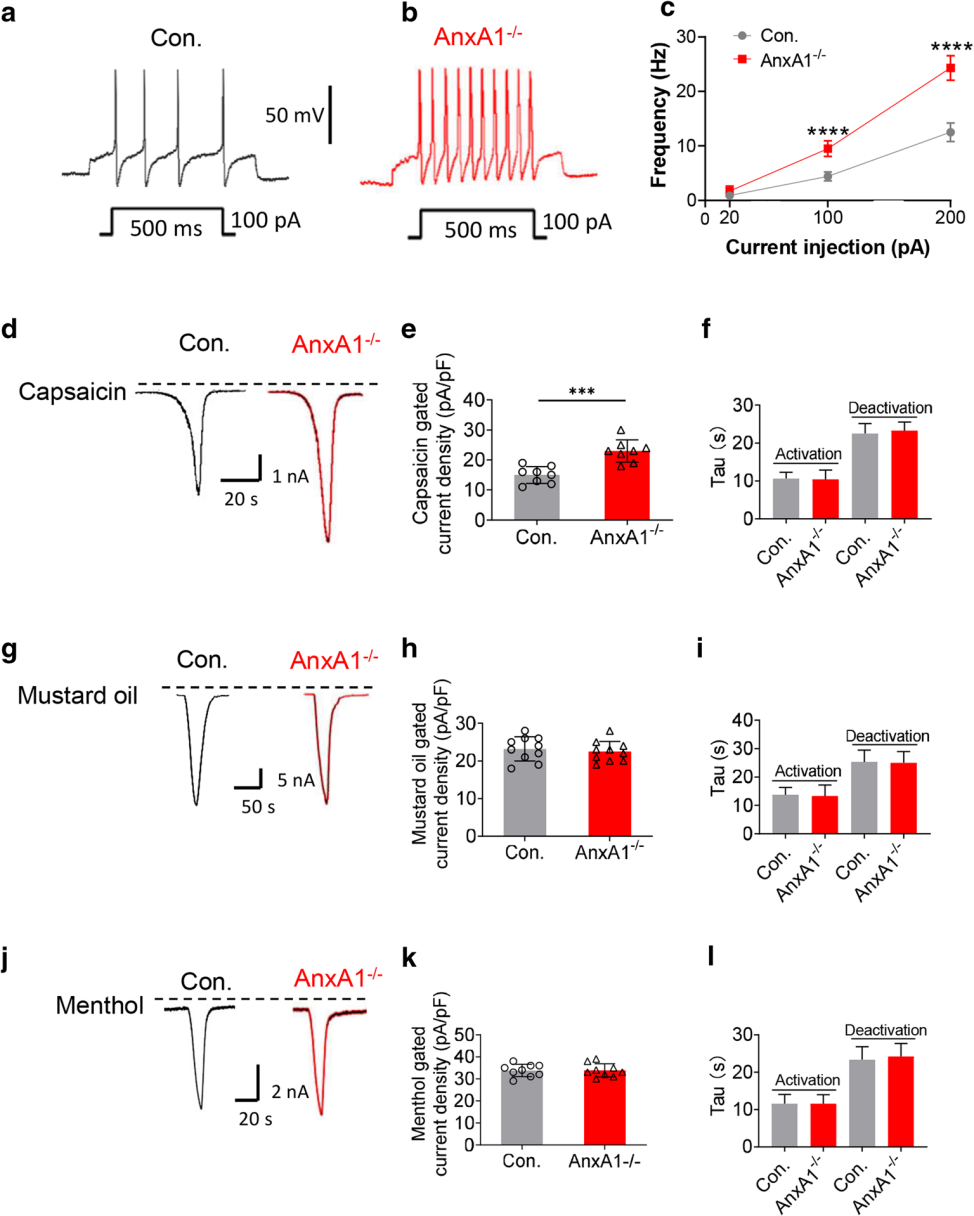
*AnxA1* deletion sensitizes capsaicin induced TRPV1 responses. Representative traces show the injected currents induced repetitive action potentials in control (**a**) and *AnxA1*^−/−^ (**b**) DRG neurons. **c** Quantification of action potential frequencies in control and *AnxA1*-deficient DRG neurons after different currents injection (20, 100, and 200 pA). (*AnxA1*^*−/−*^ versus control group, ****P < 0.0001, Two-way ANOVA repeated measures with Tukey’s multiple comparisons test. Control group, n = 10; *AnxA1*^*−/−*^ group, n = 8). Representative traces of **d** 10 μM capsaicin-gated currents, **g** 100 μM mustard oil-gated currents, **j** 100 μM menthol-gated currents and **h** 10 μM ATP-gated currents at -70 mV in control (black trace) and *AnxA1*^*−/−*^ (red trace) DRG neurons. Quantification of average current density after capsaicin **e** (*AnxA1*^*−/−*^ versus control group, ***P < 0.001, Student’s t-test, n = 8 neurons in WT group and n = 8 neurons in *AnxA1*^*−/−*^ group from 3 cultures, respectively), mustard oil (**h**) (*AnxA1*^*−/−*^ versus control group, *P* = 0.61, Student’s t-test, n = 10 neurons from 3 cultures in each group), and menthol (**k**) (*AnxA1*^*−/−*^ versus control group, *P* = 0.94, Student’s t-test, n = 9 neurons from 3 cultures in each group), application measured at the current peak between control and *AnxA1*^*−/−*^ group. Time constants Tau of capsaicin (**f**), mustard oil (**i**), and menthol (**l**)-induced activation and deactivation at -70 mV in control and *AnxA1*-deficient DRG neurons (*AnxA1*^*−/−*^ versus control group, capsaicin: *P* = 0.81, *P* = 0.55; mustard oil: *P* = 0.79, *P* = 0.87; menthol: *P* = 0.99, *P* = 0.59,  Student’s t-test). All data are represented as mean ± SD

To further verify *AnxA1* deletion enhances the sensitivity of DRG neurons, we investigated whether *AnxA1*^−/−^ could affect the intrinsic firing properties of DRG neurons by using whole-cell current-clamp recording. *AnxA1*^−/−^ cultures fired more repetitive action potentials than the control neurons after injection of 100 pA or 200 pA current (100 pA: 11.8 ± 6.6 *vs*. 5.9 ± 3.4 Hz; 200 pA: 24.3 ± 11.48 *vs*. 12.5 ± 5.9 Hz; n = 10, *P* < 0.0001; Fig. 4a–c). However, both the resting membrane potential (59.7 ± 1.9 and 60.2 ± 2.1 mV for control and *AnxA1*^−/−^ neurons, respectively, n = 10 neurons/group) and the amplitude of action potentials were not significantly changed (61.6 ± 2.3 and 59.6 ± 2.6 mV for control and *AnxA1*^−/−^ neurons, respectively, n = 10 neurons/group) (*P* = 0.26; Fig. 4a–c).

To determine whether the increased neuronal activity caused by *AnxA1* deletion is attributable to the increased TRPV1 sensitivity, we then recorded the DRG neurons under voltage clamp. Bath application of capsaicin (10 μM) elicited typical inward TRPV1 currents in both control and *AnxA1*^−/−^ cultures (Fig. 4d). However, the *AnxA1*^−/−^ group displayed larger TRPV1 current trace and significantly higher capsaicin gated current density than the control group (23.0 ± 3.7 *vs*. 15.0 ± 2.8 pA/pF; n = 8, *P* < 0.001; Fig. 4d, e), although the activation and deactivation time constants were not changed between the two genotypes (*P* = 0.81; *P* = 0.55; Fig. 4f). We also applied mustard oil (100 μM) and menthol (100 μM) to the cultures and then recorded the evoked currents. Neither TRPA1 nor TRPM8 current traces were obviously altered in the DRG cultures of the two genotypes (Fig. 4g, j). Similarly, no significant difference was observed in mustard oil and menthol gated current density between the two groups (*P* = 0.61; *P* = 0.94; Fig. 4h, k). In addition, neither activation nor deactivation time constants of TRPA1, or TRPM8 were affected in *AnxA1*^−/−^ DRG neurons as compared with control group (*P* = 0.79; *P* = 0.87; Fig. 4i; *P* = 0.43; *P* = 0.59; Fig. 4l). Together with the calcium imaging results, these findings consistently demonstrate that *AnxA1* deletion increases DRG neuronal excitability, and sensitizes responses to TRPV1.

### ANXA1 mimic peptide Ac2-26 increases intracellular Ca^2+^ and activates PLCβ via FPR2

**Fig. 5:**
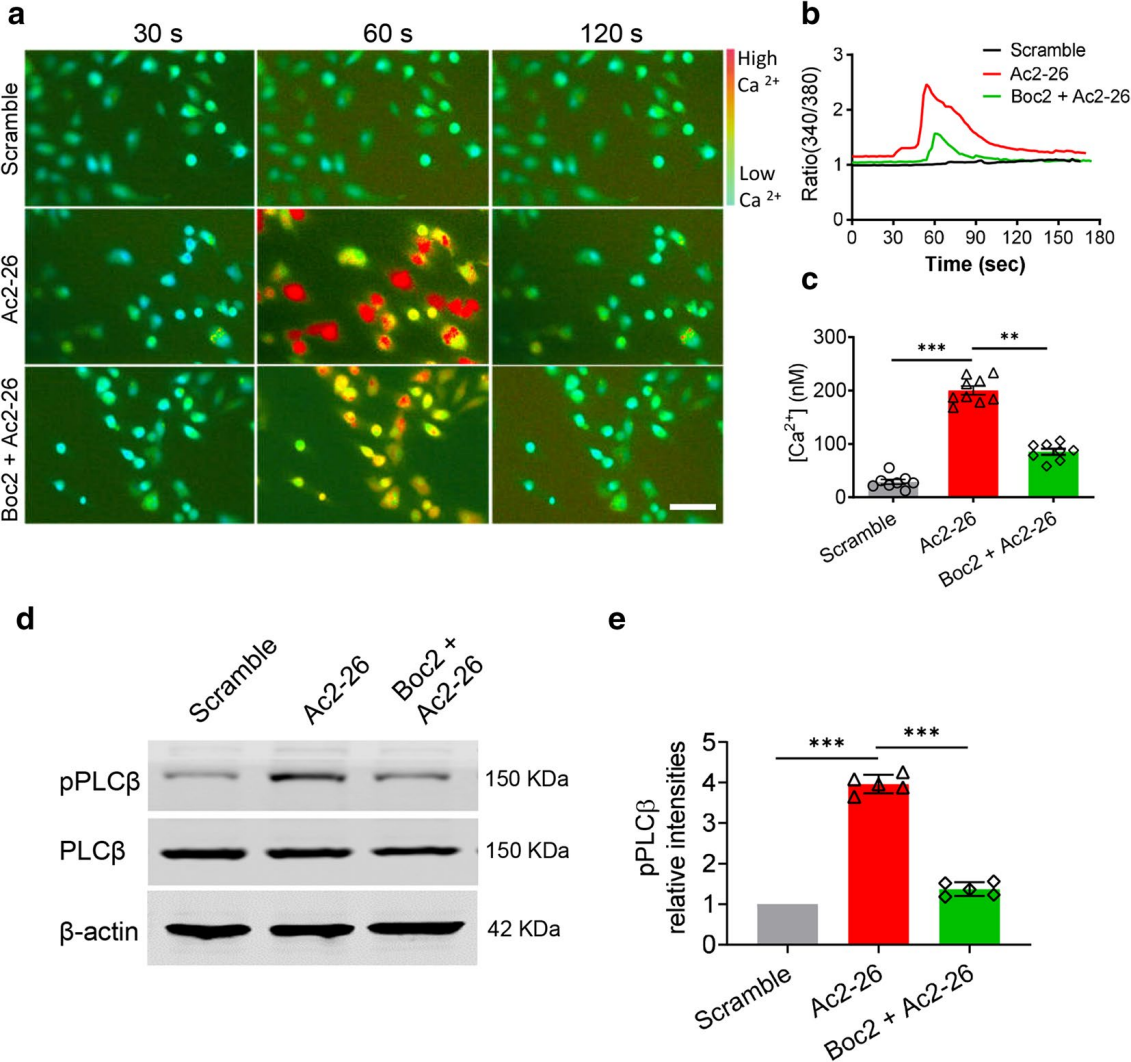
ANXA1 mimics Ac2-26 increases intracellular Ca^2+^, and activates PLCβ via FPR2. **a** Representative images of the calcium-dependent fluorescence 30, 60 and 120 s after application with scramble (3.3 μM), Ac2-26 (3.3 μM) and Boc2 (10 μM) + Ac2-26 (3.3 μM) in cultured *AnxA1*^*−/−*^ DRG neurons. Green color indicates basal intracellular Ca^2+^ concentration (i.e. before depolarization) in Fura-2 dye loaded DRG neurons. Warmer colors (yellow, orange and red) indicate that the cytoplasmic Ca^2+^ concentration is relatively higher. Scale bar, 100 μm. **b** Representative traces showing the time course of changes in ratio (340/380) of calcium-dependent fluorescence during application of scramble (3.3 μM), Ac2-26 (3.3 μM) and Boc2 (10 μM) + Ac2-26 (3.3 μM). **c**, Quantification of the intracellular Ca^2+^ concentration ([Ca^2+^]) responding to scramble (3.3 μM), Ac2-26 (3.3 μM) and Boc2 (10 μM) + Ac2-26 (3.3 μM) (Ac2-26 versus scramble group, *** P < 0.001; Ac2-26 versus Boc2 + Ac2-26 group, **P < 0.01, one-way ANOVA, post hoc Student’s t test. n = 8 in scramble group, n = 9 in Ac2-26 group and n = 8 in Boc2 + Ac2-26 group). **d** Representative protein band images of the pPLCβ and PLCβ after application with scramble (3.3 μM), Ac2-26 (3.3 μM) and Boc2 (10 μM) + Ac2-26 (3.3 μM) in cultured *AnxA1*^*−/−*^ DRG neurons. β-actin was used as the internal reference protein. **e** Quantification of the relative protein intensities of pPLCβ among scramble, Ac2-26 and Boc2 + Ac2-26 groups. The data of relative intensities in scramble group were normalized to 1 (Ac2-26 versus scramble group, ***P < 0.001; Ac2-26 versus Boc2 + Ac2-26 group, ***P < 0.001, one-way ANOVA, post hoc Tukey’s multiple comparisons test, n = 5 in each group). All data are represented as means ± SD

Given that the *AnxA1* deficiency could sensitize TRPV1 in DRG neurons, we hypothesize that ANXA1 may reduce TRPV1 sensitization. Next, we applied exogenous ANXA1 mimetic peptide Ac2-26 (3.3 μM) or the scrambled peptide (Scramble, 3.3 μM) as the control into the cultured *AnxA1*^−/−^ DRG neurons. The ratiometric calcium imaging was used again to detect whether Ac2-26 influences the intracellular calcium concentration ([Ca^2+^]i). The results showed that Ac2-26 induced a remarkable increase of [Ca^2+^]i in DRG cultures as compared with scramble control (200.3 ± 23.9 *vs*. 28.9 ± 12.7; n = 9, *P* < 0.001). However, Ac2-26 co-treatment with FPR2 antagonist Boc2 (10 μM) significantly inhibited the increase of [Ca^2+^]i in cultured DRG neurons (85.3 ± 16.0 *vs*. 200.3 ± 23.9; n = 8, *P* < 0.01; Fig. 5a–c).

To elucidate whether the increase of [Ca^2+^]i is mediated by the activation of FPR2 downstream signal phospholipase C beta (PLCβ), we detected the active form of PLCβ (phosphorylated PLCβ, pPLCβ), and the total PLCβ after applying Ac2-26 or Boc2 with Ac2-26. The results showed that Ac2-26 alone robustly increased the protein level of pPLCβ, while co-application of Boc2 with Ac2-26 apparently inhibited the increase of pPLCβ (*P* < 0.001; Fig. 5d, e). Neither Ac2-26 nor Boc2 with Ac2-26 altered the protein level of total PLCβ (Fig. 5d). Since the specific inhibitor for PLCβ is not commercially available at this stage, we did not perform further experiment by using inhibitors to validate this signaling pathway. Together, these results indicate that the increase of intracellular Ca^2+^ induced by Ac2-26 is mainly mediated by FPR2 and its downstream PLCβ.

### Ac2-26 promotes CaM-TRPV1 interaction and desensitizes TRPV1 via FPR2

**Fig. 6:**
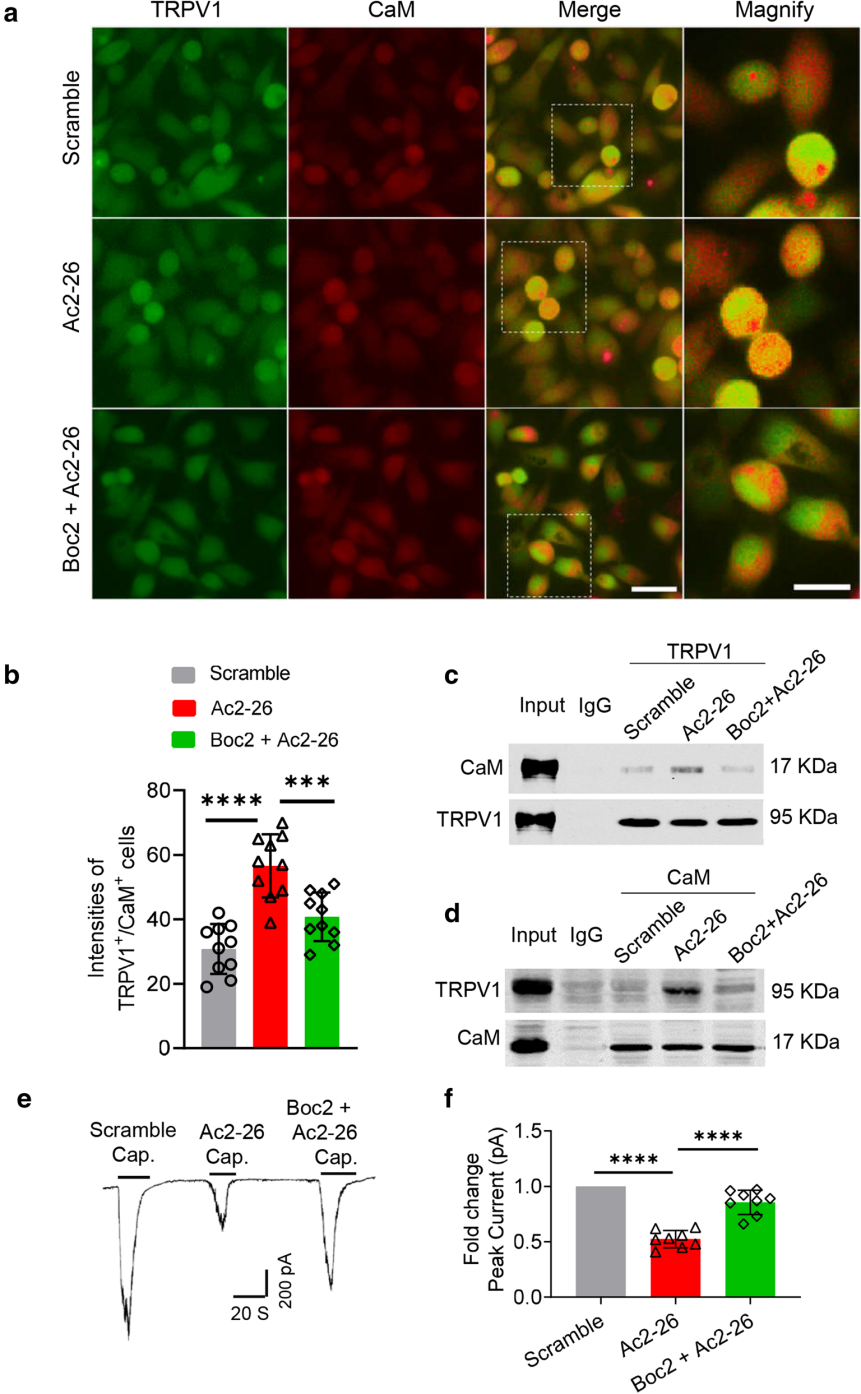
Ac2-26 enhances interaction between CaM and TRPV1 via FPR2. **a** Representative images of double immunofluorescent staining after application with scramble (3.3 μM), Ac2-26 (3.3 μM) and Boc2 (10 μM) + Ac2-26 (3.3 μM) in cultured *AnxA1*^*−/−*^ DRG neurons. The areas in the dotted box in merge pictures zoom in and present in magnify panels. Scale bar, 50 μm in TRPV1, CaM and merge pictures, 25 μm in magnify pictures. **b** Quantification of the fluorescent intensities of TRPV1 positive and CaM positive (TRPV1^+^/CaM^+^) cells among scramble, Ac2-26 and Boc2 + Ac2-26 groups. (Ac2-26 versus scramble group, ****P < 0.0001; Ac2-26 versus Boc2 + Ac2-26 group, ***P < 0.001, one-way ANOVA, post hoc Student’s t test. n = 10 in each group). All data are represented as mean ± SD. **c** and **d** Representative Western blots (WB) band images of immunoprecipitation (IP) experiments in cultured *AnxA1*^*−/−*^ DRG neurons treated with scramble (3.3 μM), Ac2-26 (3.3 μM) and Boc2 (10 μM) + Ac2-26 (3.3 μM), respectively. **c** TRPV1 antibody incubated immunoprecipitations were detected with CaM and TRPV1 antibodies by western blotting. **d** CaM antibody incubated immunoprecipitants were detected with TRPV1 and CaM antibodies by western blotting. Input, 10 μg protein of the extracts without IP was loaded. IgG, immunoprecipitants incubated with nonspecific IgG (IgG) as negative control. **e** Representative traces of capsaicin-evoked currents with co-application of scramble, Ac2-26 and Boc2 + Ac2-26 in cultured *AnxA1*^*−/−*^ DRG neurons. **f** Quantification of the fold change peak current in scramble, Ac2-26 and Boc2 + Ac2-26 groups. The data of fold change peak current in scramble group were normalized to 1 (Ac2-26 versus scramble group, ****P < 0.0001; Ac2-26 versus Boc2 + Ac2-26 group, ****P < 0.0001, one-way ANOVA, post hoc Tukey’s multiple comparisons test, n = 8 in each group)

CaM is activated once it binds to Ca^2+^ and functions as part of a calcium signal transduction pathway by influencing its interactions with target proteins. It is known that the intracellular domain (amino acids 767–801 of COOH-terminal region) of TRPV1 binds CaM in a Ca^2+^-dependent manner [11]. Therefore, we deduce that Ac2-26-FPR2 induced increase in intracellular Ca^2+^ promotes CaM-TRPV1 binding in DRG neurons. To prove this, we used double immunofluorescence staining to detect the interaction of TRPV1 and CaM in cultured DRG cells. The staining results showed that Ac2-26 obviously increased the co-localization of TRPV1 and CaM (intensities of TRPV1^+^/CaM^+^ cells) in DRG neurons as compared with scramble control (56.6 ± 9.8 *vs.* 30.8 ± 7.7, n = 10, *P* < 0.0001; Fig. 6a, b). However, co-application of Boc2 with Ac2-26 reversed the increasing of TRPV1^+^/CaM^+^ intensities in DRG cells (40.8 ± 7.5 vs. 56.6 ± 9.8; n = 10, *P* < 0.001; Fig. 6a, b). To further confirm these results, we performed co-immunoprecipitation experiments and found that Ac2-26 promoted the interaction between TRPV1 and CaM. And the enhanced interaction between the two proteins was markedly attenuated by co-treatment of Boc2 with Ac2-26 (Fig. 6c, d).

The Ca^2+^-CaM/TRPV1 interaction was found to reduce TRPV1 channel open probability^15^ and desensitize TRPV1 in a calcium dependent manner [14]. Therefore, we speculate that Ac2-26-FPR2 signal induced CaM-TRPV1 interaction may lead to the desensitization of TRPV1, ultimately decreasing nociceptive transmission at the DRG level. Subsequently, we used capsaicin to activate TRPV1 in *AnxA1*^−/−^ DRG neurons. The patch clamp recording results showed that capsaicin (Cap., 100 nM) elicited an obvious inward current in *AnxA1*^−/−^ DRG neurons. However, exposure to Ac2-26 (3.3 μM) produced an apparent decrease in the capsaicin activated current (0.5 ± 0.1 *vs*. 1.0 ± 0.0; n = 8, *P* < 0.0001; Fig. 6e, f). After co-application of Boc2 (10 μM) with Ac2-26 (3.3 μM), the effect of Ac2-26 on TRPV1 current was markedly reversed (0.8 ± 0.1 *vs*. 0.5 ± 0.1; n = 8, *P* < 0.0001; Fig. 6e, f). In addition, we found a similar desensitized effect of Ac2-26 on TRPV1 current via FPR2 in wild type DRG neurons (Ac2-26 *vs.* Scramble: 0.5 ± 0.1 *vs*. 1.0 ± 0.0; Boc2 + Ac2-26 vs. Ac2-26: 0.9 ± 0.2 *vs*. 0.5 ± 0.1; n = 10, *P* < 0.0001; Additional file 3a, b).

The desensitization of TRPV1 caused by Ac2-26-FPR2 signal was further confirmed in vivo on *AnxA1*^−/−^ mice with inflammatory pain. In formalin mice model, intrathecal injection of Ac2-26 (1 mg/kg, 10 μl, 1 h before the formalin injection) decreased the licking and biting time by 45–50% in the first peak of nociception (62.6 ± 12.1 *vs.* 109.0 ± 17.1; n = 8, *P* < 0.01; Additional file 4a) and 30% in the second peak compared to scramble control (213.0 ± 20.6 *vs.* 317.0 ± 36.4; n = 8, *P* < 0.001; Additional file 4a). However, the anti-nociceptive effect was reversed by co-application of the FPR2 antagonist Boc2 (10 mg/kg, 10 μl) with Ac2-26 (0–15 min: 91.9 ± 17.1 *vs.* 62.6 ± 12.1; n = 8, *P* = 0.02; 15–60 min: 280.6 ± 29.3 *vs.* 213.0 ± 20.6; n = 8, *P* < 0.01; Additional file 4a). Similarly, In CFA induced inflammatory pain, intrathecal Ac2-26 application (1 mg/kg, 10 μL, 12 h and 1 h before the formalin injection) significantly increased the thermal pain latency by 30–40% (9.6 ± 1.8 *vs.* 6.7 ± 1.1; n = 10, *P* < 0.01; Additional file 4b), which was abolished by co-administration of Boc2 with Ac2-26 (7.3 ± 0.8 *vs.* 9.6 ± 1.8; n = 10, *P* < 0.01; Additional file 4b). Overall, these results suggest that Ac2-26 promotes CaM-TRPV1 interaction and desensitizes TRPV1 via FPR2/ALX, and finally exerts anti-nociceptive effects in inflammatory pain at the DRG level.

## Discussion

In this study, we show that genetic deletion of ANXA1 selectively promotes thermal and capsaicin induced hyperalgesia by enhancing TRPV1 function in dorsal root ganglion neurons. Furthermore, ANXA1 mimic peptide Ac2-26 desensitizes TRPV1 through FPR2-PLCβ-Ca^2+^-CaM signaling at the DRG level. Our study reveals the mechanism by which ANXA1 exerts antinociceptive effect in inflammatory pain.

ANXA1 is a glucocorticoid mediated anti-inflammatory protein that is encoded by the *AnxA1* gene [12] and involved in innate and adaptive immunity [13], cancer diseases [14], and inflammation [15] Early studies found ANXA1 peptidomimetics have analgesic effects in inflammatory pain both peripherally and centrally [16, 17]. Our previous study in rats also have shown the analgesic effects of Ac2-26 in CFA induced inflammatory pain at the DRG level [5]. In addition, ANXA1 inhibits remifentanil-induced hyperalgesia via regulating spinal CXCL12/CXCR4 in rats [18]. Here we found that deletion of ANXA1 enhances noxious sensations to capsaicin, thermal stimuli and inflammation, which is consistent with Ayoub’s discovery that global *AnxA1* knockout mice are more vulnerable to inflammatory visceral pain induced by intraperitoneal acetic acid injection than wild-type mice [4].

Based on the results that *AnxA1* deficiency selectively increases heat or capsaicin, but not cold, mechanical, mustard oil or menthol induced nociception, we assume that deletion of *AnxA1* may specifically affect the functions of TRPV1 instead of TRPA1 or TRPM8. Indeed, *AnxA1* knockout had no effect on TRPV1 expression, but increased the sensitivity of the TRPV1 ion channel, as shown by calcium imaging and whole cell patch clamp recordings. Despite the fact that ANXA1 and TRPV1 co-localize in DRG neuronal cells, there is no clear evidence demonstrating their direct interaction.

Our results show 10 μM capsaicin-induced robust Ca^2+^ response in *AnxA1*^−/−^ DRG neurons as compared with control. In this case, capsaicin directly activates TRPV1, which causes basal calcium influx. Once the *AnxA1* is deleted, TRPV1 becomes more sensitized under capsaicin stimulation, and this non-selective cation channel opens more frequently, which leads to plenty of extracellular Ca^2+^ influx and results in a greater Ca^2+^ response. We also found 3.3 μM Ac2-26 induced dramatic increase of Ca^2+^ signal when compared with scramble peptide in *AnxA1*^*−/−*^ DRG neurons. In this situation, Ac2-26 directly activates FPR2 and the downstream PLCβ, which increases the second messenger IP3 in the cytoplasm. It is known that IP3 interacts with IP3 receptor and facilitates calcium release from endoplasmic reticulum stores, thus causing a large increase in intracellular Ca^2+^ concentration and producing a significant increase in Ca^2+^ response. This result is consistent with a study in polymorphonuclear cells showing that the activated heterotrimeric Gi/o-protein of FPR2 by ANXA1 dissociates into α and βγ subunits, activating downstream PLCβ and phosphoinositide 3-kinase (PI3K). Activated PLCβ converts phosphatidylinositol 3, 4, 5-triphosphate (PIP3) into diacylglycerol (DAG) and inositol-1, 4, 5-triphosphate (IP3), which facilitates calcium release from endoplasmic reticulum stores [19–22]. Overall, these studies suggest that ANXA1-FPR2 signaling is conserved and plays a pivotal role in anti-inflammation and pro-resolution of inflammation, corroborating our previous findings that ANXA1 inhibits CFA induced inflammatory pain in rats via FPR2 in DRGs [5].

The most significant finding of this study is that ANXA1-FPR2 signaling increases intracellular calcium and activates CaM, which interacts with TRPV1, decreases TRPV1 sensitivity and relieves nociception. Although intracellular Ca^2+^ is necessary for neuronal excitation and synaptic transmission, excessive intracellular Ca^2+^ can decrease cellular excitability [23]. For example, higher Ca^2+^ may prevent sodium from moving through voltage-gated sodium channels, resulting in reduced depolarization and action potential generation [24]. Furthermore, the release of calcium from internal stores can reduce neuron excitability [25] suggesting that drugs that cause calcium release from stores may also affect neuronal excitability. In Xenopus oocytes and HEK 293 cells, Ca^2+^ and CaM form a complex of Ca^2+^/CaM that binds to the intracellular NH2-terminal domain of TRPV1 and inhibits gating to reduce channel open probability [10]. As a result, the significantly elevated Ca^2+^ in DRG sensory neurons that express TRPV1 can cause channel closure and desensitization to noxious sensory stimuli. In addition, the structure of TRPV1’s cytosolic ankyrin repeat domain (ARD) has been identified as a CaM binding site, which is essential in channel sensitivity regulation [26]. Thus, desensitization of TRPV1 by ANXA1/FPR2 via downstream Ca^2+^/CaM/TRPV1 may represent a feedback mechanism that not only protects the cell from excitotoxicity of excessive Ca^2+^, but also contributes to the analgesic effects of Ac2-26.

## Conclusions

In conclusion, we showed that selective deletion of *AnxA1* in DRG neurons enhanced noxious heat or capsaicin induced nociception, increased capsaicin-mediated Ca^2+^ response, and enlarged capsaicin-induced TRPV1 current in mice DRG neurons. ANXA1 mimic peptide Ac2-26 can increase intracellular Ca^2+^, activate CaM, promote the interaction between CaM and TRPV1, desensitize TRPV1 through FPR2, and finally reduce nociceptive transmission and exerts analgesic effects (Additional file 5).

## Materials and methods

### Generation of ***AnxA1*** conditional knockout (***AnxA1***^−/−^) mice.

The exon 6 of the *AnxA1* targeting construct was flanked by *loxP* sites followed by a FRT-flanked NEO cassette. The targeting vector was inserted into r1 embryonic stem cells (129/Sv, ThermoFisher Scientific) with electroporation. Neomycin-resistant cells were selected with geneticin (G418 Sulfate, ThermoFisher Scientific, Cat# 10,131,035). The confirmed positive clones were injected to blastocysts of C57BL/6 mice, which produced substantial chimeric mice. To generate heterozygous mice with one *loxP*-flanked allele (*AnxA1*^*loxP/*+^), the chimeric mice were bred with C57BL/6 mice. Next, the NEO cassette was deleted by crossing *AnxA1*^*loxP/*+^ mice with FLP deleter mice (B6.129S4-*Gt(ROSA)26Sor*^*tm1(FLP1)Dym*^/RainJ, Jackson Laboratory, stock 009,086). To specifically delete *AnxA1* gene in DRG, NEO cassette deficient *AnxA1*^*loxP/*+^ mice were crossed with *Avil*^*cre*^ mice to produce *Avil*^*cre*^; *AnxA1*^*loxP/*+^ mice, which were then crossed with *AnxA1*^*loxP/*+^ mice to generate ANXA1 conditional knockout (*AnxA1*^−/−^) mice (*Avil*^*cre*^; *AnxA1*^*loxP/loxP*^). The littermates (*Avil*^*cre*^-*AnxA1*^+/+^) with the same age and sex were used as control. All mice were genotyped two weeks after birth, using reverse-transcription PCR with specific primers (forward, 5’- GGTGTGAATGAAGACTTGGCTGA-3’ and reverse, 5’- GTTTCATCCAGGATGGCTTGGCA -3’).

All the animals were fed on a standard chow pellet diet with tap water ad libitum in the standard conditions (22 ± 2℃ temperature, 40–60% humidity) with a 12 h light/dark cycle at the Specific-Pathogen-Free (SPF) facility. All animal procedures were conducted with the approval of the Animal Ethics Committee of Huazhong University of Science and Technology and with the guidelines of Editorial by Drummond JC and Guide for the Care and Use of Laboratory Animals [27, 28] Sample sizes of 8 to 10 mice per group were used in each animal experiments.

### Reagents

The ANXA1 mimetic peptide and the scrambled control peptide ANXA1 2–26 (Ac2-26, acetyl-AMVSEFLKQAWFIENEEQEYVQTVK) and the scramble peptide (acetyl-YESQFKAVWVE-INTQQMLKFEAEEV) were generated from GenicBio Limited (Shanghai, China) by using solid-phase stepwise synthesis. The FPR2 antagonist N-t-Boc-Phe-Leu-Phe-Leu-Phe (Boc2) were purchased from Calbiochem (San Diego, CA, USA). Other materials were obtained from Sigma-Aldrich (Saint Louis, US). Ac2-26 and Boc2 were applied into the bath solution of cultured DRG neurons in a volume of 10 μL. When co-administered Boc2 with Ac2-26, we applied the first single-drug injection combined with the equivalent vehicle volume of the second drug. Drugs were freshly dissolved in saline each test day. Boc2 stock solutions were prepared by dissolving the compounds in dimethylsulphoxide (DMSO); aliquots of this solution were used for subsequent dilution in saline (DMSO: saline 1:3, v/v). We chose these doses based upon our preliminary experiments.

### Thermal and mechanical pain behavior measurements

Thermal and mechanical was measured according to our previous protocol [5, 29] and the operations of the algometer follows the procedures of the manual guide (Ugo basil). All the behavioral experiments were conducted starting at 9:00 AM every day. Before the experiments, animals were acclimatized for 30 min in a transparent Plexiglas box in the room for pain measurements. Each mouse was tested three times, with intervals of 10 min (Please see supplementary materials for the detailed information).

1. **Hot plate test**
  The hot plate used in this study was a Series 8 Model 39 (IITC Life Sciences, US). The temperature in the hot plate was set to 50℃ with a cut-off time of 45 s and typically left for at least 30 min in order to prevent the plate temperature from fluctuating beyond ± 0.1 °C during the experiment. Mice were placed on the hot plate as gently as possible with the experimenter using a foot switch to activate the internal timer with accuracy up to a hundredth of a second. We used the paw licking as the readout of pain response. Use the time of onset of pain response as the pain latency.
2. **Hargreaves test**
  The Hargreaves test instrument used in this study was a BW-Plantar 390 (IITC Life Sciences, US). A radiative heat source was placed beneath the animal and pointed at the plantar surface of the hindpaw. The time between the onset of the thermal radiation stimulus and the appearance of paw withdrawal was recorded as the hindpaw withdrawal thermal latency.
3. **Von Frey test**
  Classical top-down methods [30] were used to detect the mechanical threshold of the mice hindpaw withdrawal to the von Frey filaments (Product #58,011; Set of 20 monofilaments: Stoelting Co., Wood Dale, IL, USA) stimuli. Mice under test were placed on the metal grid with pores (2 mm × 2 mm) and separated with polyethylene chamber (8 cm × 8 cm × 15 cm). After waiting for the mice to adapt to the chamber, using von Frey filaments with different force intensity to poke the hindpaw skin area between the third and four toes. Stimulus intensity typically starts at 0.4 g. If the filament bent more than 90 degrees and the mouse still do not lift their feet, it will be considered unresponsive. Then, adjacent filaments with a higher stimulus intensity should be given. If there is a response, adjacent filaments with a lower stimulus intensity should be replaced. Each stimulation interval must be longer than 10 s until a filament is found that causes a 50% lift-off response. The maximum stimulation force was 4.0 g. Record the stimulation intensity that could cause 50%-foot lifting response in the tested mice as the mechanical threshold of the hindpaw withdrawal.
4. **Cold plate test**
  Before the cold plate test, set plate (Series 8, PE34, IITC Life Sciences, US) temperature to match that of the test room (25 ± 2℃), clean test plate surface and place mice into the chamber on the plate to acclimate for 30 min. The metal plate was cooled down to 4 °C and the time of the onset of pain behavior such as lifting, shaking, licking or jumping of the paws is defined as the response latency. As soon as the behavioral response is seen, quickly remove the mouse from the test surface to avoid unnecessary suffering or tissue damage. After finishing the test, remove mouse into a separate cage from untested mice. Clean any urine or feces from the plate surface, and return the test plate temperature to that of the room and clean surface in preparation for next mouse.
5. **Randall-Selitto test**
  Using a Paw Pressure Test Apparatus (Part# 2500, IITC Life Sciences, US) to perform the Randall-Selitto test. Before testing, mouse was placed into the restrainer for habituation until its breathing is normal and mouse is not agitated. Then the mouse’s tail was placed onto the pedestal of the Randall-Selitto apparatus. A point on the tail approximately one quarter of the way down from the base of the tail was chosen so that the animal could withdraw the tail easily. Pressure was applied to the foot pedal to increase the weight exerted onto the tail. The foot pedal was released and the blunt cone was lifted at the first sign of struggling, vocalization, or withdrawal of the tail. The number reached on the scale was recorded and multiplied by the weights to obtain the final force exerted on the tail.

### Chemical nociception measurements

A week before the test, gently handling the mice for several times to reduce animals’ stress response. On the testing day, mice were placed into the transparent Perspex box for habituation until its exploratory behavior has ceased. Then took the mice from the box for injection. Insert the 0.3 ml disposable insulin syringe needle into the center of the hindpaw at a shallow angle and subcutaneously inject the indicated dose of different chemicals (capsaicin (500 µg/ml, Sigma-Aldrich), 5% mustard oil (Sigma-Aldrich), menthol (0.8 mg/ml, Fisher Scientific), or 2% formalin (Fisher Scientific) in a total volume of 50 μl dissolved in saline). The mice should not bleed during or after injection. Finally, the mice were placed back into the Perspex box and use the stopwatch to record the time spent conducting nocifensive behaviors (licking or biting) for the desired amount of time (Menthol, mustard oil, capsaicin for 5 min and formalin for 60 min, respectively).

### Complete Freund’s adjuvant (CFA) and nociception tests

Mice were anesthetized with 1% isoflurane and injected with 20 μl of Vehicle (saline, buffered with 20 mM HEPES, pH 7.4) or complete Freund’s adjuvant (CFA, 0.5 mg/ml heat-killed M. tuberculosis; Sigma, St. Louis, MO) in the plantar surface of the hind paw to induce chronic inflammation. Based on our previous work,^5^ the Hargreaves test and von Frey test described above are used to assess thermal hyperalgesia and mechanical allodynia on day 7 after CFA injection. Please see supplementary materials for the detail information.

### Acute isolation and culture of DRG neurons

Both *AnxA1*^−/−^ mice and control littermates were euthanized by asphyxiation method (CO_2_) followed by decapitation. L4, L5 and L6 DRGs were quickly removed, carefully dissected and placed in ice-cold Ringer's solution (130 mM NaCl, 3 mM KCl, 2 mM CaCl_2_, 2 mM MgSO_4_, 1.25 mM KH_2_PO4, 26.2 mM NaHCO_3_, and 10 mM dextrose and filled with 95% O_2_ and 5% CO_2_, pH = 7.4, osmolality of 290–310 mOsm). They were digested with calcium-free standard Ringer's solution containing 1.0% collagenase type II (collagenase, 265 U/mg; Worthington Biochemical Corp., Lakewood, NJ) for 30 min at 37℃ and then washed in Ringer's solution for 10 min. DRG cells were dissociated by pipetting with a polished Pasteur dropper and transferred to polynaphthylamine-coated coverslips containing Medium 199 (Sigma Chemicals Co., St. Louis, MO) with 10% fetal bovine serum and 1,000 U/ml penicillin and streptomycin. DRG cells were cultured in a 37 °C incubator (containing 5% CO_2_).

### Calcium imaging

Calcium imaging was performed 48 h after plating the cells. Intracellular [Ca^2+^]i labeling was performed using the Ca^2+^-sensitive fluorescent dye fura-2. Indicators were applied by adding 5 μM fura-2 acetoxymethyl ester to DRGs neurons for 45 min at 37° C. Fura-2 acetoxymethyl ester dissolve in HEPES-buffered Hanks' saline solution (HHSS, pH 7.45, in mM): 20 HEPES, 137 NaCl; 1.3 CaCl_2_, 0.4 MgSO_4_, 0.5 MgCl_2_, 5.4 KCl, 0.4 KH_2_PO_4_, 0.3 Na_2_HPO_4_, 3.0 NaHCO_3_ and 5.6 glucose) with 0.5% bovine serum albumin (BSA). Subsequently, the coverslips with attached cells were placed in the detection chamber of the inverted microscope (Olympus IX70 microscope, Olympus Optical, Tokyo, Japan). Before the experiment, HHSS was continuously filled in the chamber at a rate of 1.0 to 1.5 ml/min for 10 min. Ca^2+^ signal was detected via a 40 × objective of the microscope (UApo/340, numerical aperture = 1.35). Fura-2 digital images were acquired by Optoscan monochromator (Cairn Research LTD, Faversham, Kent, UK) with fast conversion at 340 nm (8 nm bandwidth) and 380 nm. Emitted light at 517 (30) nm (Roper, Tucson, AZ) was detected using a step-cooled charge-coupled camera. Images were analyzed using Metafluor image software (Molecular Devices, Sunnyvale CA). The fluorescence change was converted to [Ca^2+^]i after calculation by the formula [Ca^2+^]i = Kdβ (R-Rmin)/(Rmax-R). R represents the fluorescence ratio of 340/380 nm. Kd represents the dissociation constant of 140 nM fura-2, and β represents the ratio of the 380 nm fluorescence emission light in the absence of Ca^2+^ to 380 nm fluorescence emission light in the presence of Ca^2+^. Rmin, Rmax, and β were determined by Ca^2+^-free buffer enomycin (1 mM EGTA) and saturated Ca^2+^ buffer (5 mM Ca^2+^) in the cell bath, and the values of Rmin, Rmax, and β were 0.237, 4.10, and 6.52, respectively. The composition of the internal solution containing EGTA was as follows (in mM): CsCl 150, MgATP 5.0, Li_4_GTP 0.1, EGTA 10, CaCl_2_ 3.0 (free Ca^2+^ concentration ∼100 nm) as well as HEPES 10 (pH 7.2 adjusted with CsOH).

### Whole-cell patch-clamp recordings

TRPV1 currents were recorded using an Axopatch 200B ultra-low-noise patch-clamp amplifier (Axon Instruments, USA). DRG neurons were placed in an open recording bath filled with Ringer's solution (mM: 140 NaCl, 5 KCl, 2 CaCl_2_, 1 MgCl_2_, 10 HEPES, and 10 glucose, pH 7.4, 21 °C). Glass electrodes (recording electrodes, 1.65 ± 0.05 μm outer diameter) were pulled with an electrode puller and filled with an internal solution of electrodes (mM: 140 KCl, 5 MgCl_2_, 4 Na_2_ATP, 0.3 Na_3_GTP, 2.5 CaCl_2_, 5 EGTA, and 10 HEPES, pH 7.2) with an electrode resistance of 2–4 MΩ before recording. In whole-cell mode, the current and voltage of neurons were maintained at 0 and less than − 45 mV for 5 min, respectively. Capsaicin (10 μM), Mustard oil (10 mM), Menthol (100 μM), ATP (10 μM), Ac2-26 (10 μM), Boc2 (100 μM) (the antagonist of FPR2), and saline (vehicle control) were applied to adjacent recording cells through a gravity-driven drug-loading perfusion system. All testing was performed at approximately 22 °C. The clamp voltage (Vh) was set to − 60 mV for all experiments, and capsaicin evoked currents were recorded in voltage clamp mode. Divide the whole-cell current by the cell capacitance to calculate the current density. Capsaicin evoked whole-cell currents were filtered and the signal sampling frequency was 1 kHz. Membrane potentials were expressed as absolute values (millivolts, mV), and TRPV1 currents were expressed as absolute values (nanoamperes, nA) or multiples of changes compared with basal values. To detect neuronal excitability, the rheobase was measured in current-clamp mode by input step currents (100 pA for 500 ms). The rheobase was reflected by the change in the basal value of the stimulus current intensity (normalized to the basal value 1) and expressed as a percentage.

### Double immunofluorescent staining

Mice were deeply anesthetized with 5% isoflurane and transcardially perfused with normal saline, followed by cold (4 °C) 4% paraformaldehyde (0.01 M PBS, pH 7.4). The L4-6 DRGs were removed, postfixed in 4% paraformaldehyde overnight, and then treated with 30% sucrose (in 0.1 M PB, pH 7.4) till tissues sink to the bottom of the container. Cryosections (10 μm) were cut, mounted on slides, and stored at − 20 °C. For immunofluorescent staining, DRG sections were treated with 10% TritonX-100 for 30 min. Nonspecific binding sites were blocked with 5% fetal bovine serum for 30 min after washing with PBS for 10 min, followed by incubation with rabbit polyclonal antibody against TRPV1 (1:500, Cat#ab6166, Abcam), rabbit polyclonal antibody against TRPA1 (1:2000, Cat#OST00061W, Invitrogen), rabbit polyclonal antibody against TRPM8 (1:1000, Cat#OSR00077W, Invitrogen), mouse monoclonal antibody against FPR2 (1:1000, Cat#H00002358-M02, Abnova), and mouse monoclonal antibody against Calmodulin (1:1000, Cat#05-173, Sigma-Aldrich), overnight at 4 °C. Then, the sections were washed with PBS for three times and 5 min each, and incubated (room temperature, 1 h) with FITC-labeled donkey anti- rabbit antibody (1:2000; Cell Signaling) and Cy3-labeled donkey anti-mouse antibody (1:2000; Cell Signaling). After cover the sections with fluorescent bleach-proof mounting medium and cover slides, fluorescent signal was detected using a Zeiss confocal fluorescence microscope (ZEISS510 META, Germany). Image J software (Bethesda, MD, USA) was used to analyze the acquired images. For immunofluorescent staining with cultured DRG cells, the cells were first rinsed with pre-warmed (37 °C) PBS, and then fixed with cold (4 °C) 4% paraformaldehyde for 20 min and then washed three times with PBS for 5 min each. The rest of the procedures were the same as for DRG sections. All immunoreactive positive profiles in a section or cultured dish were outlined, creating an artificial overlay. Counting of double-labelled cells was conducted on 6 confocal images randomly taken from two view fields from each section or cultured dish with Image J software.

### Coimmunoprecipitation

Cultured cells were homogenized with cold lysate containing 0.1% SDS, 50 mM Tris–HCl, 150 mM NaCl, 1% NP-40, 2 mM EDTA, 0.5% sodium deoxycholate, 1% Triton X-100, 1% deoxycholate, and 1 mM phenylmethylsulfonyl fluoride (PMSF), followed by rapid sonication and centrifugation at 12,000 g for 30 min at 4℃ to collect the lysate. Protein concentration was measured with the Bradford assay (Bio-Rad, Hercules, CA). The lysate was incubated with non-specific IgG (2 mg, Sigma-Aldrich, Cat#12-371), polyclonal rabbit anti-TRPV1 (2 mg, Alomone Labs, Cat #ACC-030), or monoclonal mouse anti-Calmodulin (2 mg, Sigma-Aldrich, Cat#05-173) overnight at 4 ℃. Then, added Protein G-Sepharose (2 mg/ml, Millipore, Cat#P3296) for 3 h at 4 ℃. The pellet was washed 4 times with lysis buffer, denatured with SDS sample buffer and proteins were separated with 12% SDS-PAGE gel. Next, the proteins were transferred to nitrocellulose membranes by wet transfer using Bio-Rad's protein electrophoresis transfer tank overnight at 4 ℃, and then the membranes were blocked with 5% non-fat milk for 1 h at room temperature. After three washes, they were incubated with rabbit polyclonal antibody against TRPV1 (1:1000, Cat#ab6166, Abcam) and mouse monoclonal antibody against Calmodulin (1:1000, Cat#05-173, Sigma-Aldrich) for 1 h at room temperature. Membranes were washed three times for 10 min with buffer of TBST and incubated with horseradish peroxidase-labeled goat anti-rabbit (1:2000, Cat#7074, Cell Signaling) and horse anti-mouse (1:2000, Cat#7076, Cell Signaling) secondary antibody for 1 h at room temperature. Lanes labeled "input" were loaded with 10% immunoprecipitated protein lysate. Finally, the band signals were detected with an enhanced chemiluminescence detection kit (Amersham Biosciences, Arlington, IL). The intensity of the band signal at the position corresponding to CaM and TRPV1 or CaM and FPR2 reflects the degree of interaction between the two proteins.

### Western blotting

Mice were euthanized by rapid cervical dislocation. The L4-6 DRGs were quickly removed and homogenized in ice-cold lysis buffer (pH 7.5, 50 mM Tris–HCl) including 0.1% cholic acid, 150 mM NaCl, 0.1% Nonidet P40, 2 mg/ml leupeptin, 2 mM EDTA, 1 mg/ml pepstatin and 2 mM phenylmethylsulfonyl fluoride. The homogenates were centrifuged at 15,000 g for 10 min at 4 °C to yield the total protein extract in the supernatant. The protein concentrations were measured according to the previously described methods.^5^ Each lysate (30 μg) was separated on sodium dodecyl sulfate polyacrylamide gel electrophoresis gels and transferred to polyvinylidene difluoride (PVDF) membranes (Bio-Rad) using a semidry electrotransfer system (Amersham Biosciences). Then, incubating the membranes with primary antibodies (rabbit polyclonal anti-ANXA1 (sc‐11387, 1:200; Santa Cruz Biotechnology, Santa Cruz, CA, USA), mouse monoclonal antibody against FPR2 (1:1000, Cat#H00002358-M02, Abnova), polyclonal rabbit anti-TRPV1 (1:1000, Cat#ACC-030, Alomone Labs), rabbit polyclonal antibody against TRPA1 (1:2000, Cat#OST00061W, Invitrogen), rabbit polyclonal antibody against TRPM8 (1:1000, Cat#OSR00077W, Invitrogen), mouse monoclonal antibody against Calmodulin (1:1000, Cat#05-173, Sigma-Aldrich), mouse monoclonal anti-PLCβ (1:500, Cat#05-164, Sigma-Aldrich) and rabbit polyclonal anti-pPLCβ (1:1000, #2481, Cell Signaling)) at 4 °C overnight. Next, the PVDF membranes were washed and incubated with corresponding secondary antibodies. To control the loading differences in total protein amounts, antibody to protein β-actin (1:5000, Cat#ABT264, Sigma-Aldrich) was used. The signal and intensity of the band on the PVDF membrane were detected using Odyssey system (Li-Cor Bioscience, Lincoln, NB, USA).

### Statistical analysis

All variance values in the study were represented as mean ± SD. Statistical analyses were conducted by the software GraphPad prism 8.0 (San Diego, CA). Two-way ANOVA with repeated measures was used to compare the data of bodyweight gain between the two genotypes. Pain behavioral results were analyzed by unpaired t-test. Western blotting data were analyzed by unpaired t-test (for two groups) or one-way analysis of variance (ANOVA) (for more than two groups) with the Student–Newman–Keuls (SNK) tests. Percentage of calcium responses were analyzed by unpaired t-test. Action potential frequencies were analyzed by two-way ANOVA repeated measures with Tukey’s multiple comparisons test. Current density and Tau were analyzed by unpaired t-test. [Ca^2+^] concentration, peak current and number of positive cells among three groups were analyzed by one-way ANOVA with the Student–Newman–Keuls (SNK) tests. *P* denotes the significance (*P < 0.05, **P < 0.01, ***P < 0.001, ****P < 0.0001) and refers to the respective control or indicated group.

## Supplementary Information

**Additional file 1**: ANXA1 is completely deleted in the DRG sensory neurons of AnxA1-/- mice. (a) Representative images of double immunofluorescence staining on cryosections of mouse L4-6 DRGs colabeled for ANXA1 and TRPV1 in control mice (Con., upper panel) and AnxA1-/- littermates (AnxA1-/-, lower panel). Scale bar, 100 μm. (b) Statistical bar graph shows the number of TRPV1 positive and FPR2 positive cells in DRG between the AnxA1-/- mice and control littermates. (c) Representative western blots band images and the quantification of the indicated proteins (d) ANXA1 (AnxA1-/- versus control group, ****P < 0.00001, Student’s t test. n=6 in WT group, n=6 in AnxA1-/- group).

**Additional file 2**: FPR2 co-expressed with TRPA1 and TRPM8 in DRG neurons of AnxA1-/- mice. (a and c) Representative images of double immunofluorescence staining on cryosections of mouse DRG co-labeled for FPR2 and TRPA1 in AnxA1-/- mice (c). (b and d) Venn diagram corresponding to the images in left panels showing the percentages of FPR2 positive and TRPA1 positive (a) or FPR2 positive and TRPM8 positive (c) neurons in DRG sections from ANXA1-/- mice as indicated. Scale bar, 100 μm.

**Additional file 3**: Ac2-26 inhibits TRPV1 currents via FPR2 in DRG neurons of wild type mice. (a) Whole-cell current responses to applications of capsaicin (Cap.; 100 nM) + scramble, capsaicin + Ac2-26 (3.3 μM) and capsaicin + Boc2 (10 μM) + Ac2-26 (3.3 μM), respectively. (b) Statistic bar graph shows the fold change peak current in (a), scramble control was normalized to 1 for comparison. (Ac2-26 versus scramble group, ****P<0.0001; Ac2-26 versus Boc2+Ac2-26 group, ****P<0.0001, one-way ANOVA, post hoc Tukey’s multiple comparisons test, n=10 in each group). All data are represented as mean±SD.

**Additional file 4**: Ac2-26 alleviates inflammatory pain via FPR2 in AnxA1-/- mice. (a) Quantitative analysis of the licking or biting duration over 60 min after injection of 1% formalin into the hindpaw of AnxA1-/- mice (0-15 min: Ac2-26 versus scramble group, n=8, **P < 0.01; Boc2+Ac2-26 versus Ac2-26 group, n=8 *P < 0.05; 15-60 min: Ac2-26 versus scramble group, n=8, ***P < 0.001; Boc2+Ac2-26 versus Ac2-26 group, n=8 **P < 0.01; Two-way ANOVA, Sidak’s multiple comparisons test, n=8 in each group). (b) Quantitative analysis of the withdrawal latency to radiant heat in Hargreaves test (Ac2-26 versus scramble group, n=10, **P < 0.01; Boc2+Ac2-26 versus Ac2-26 group, n=10, **P < 0.01; One-way ANOVA, post hoc Tukey’s multiple comparisons test) in AnxA1-/- mice treated with after unilateral injection of CFA.

**Additional file 5**: Summary graph. (Left) Genetic deletion of AnxA1 (AnxA1-/-) increases capsaicin mediated Ca2+ response and TRPV1 current in DRG neurons, and selectively enhances noxious heat or capsaicin induced pain sensation. (Right) ANXA1 mimic peptide Ac2-26 binds with FPR2, activates FPR2 coupled Gi/o signaling pathway, increases intracellular Ca2+, which binds to calmodulin (CaM) and enhances CaM-TRPV1 interaction, thus desensitizes TRPV1, finally decreases the nociceptive transmission and exerts analgesic effects.

## Data Availability

The datasets used and/or analyzed during the current study are available from the corresponding author on reasonable request.

## Abbreviations

ANXA1: Annexin A1
FPR2/ALX: Formyl peptide receptor 2/receptor for lipoxin A4
Ac2-26: ANXA1 2–26
Boc2: N-t-Boc-Phe-Leu-Phe-Leu-Phe
DRG: Dorsal root ganglia
TRPV1: Transient receptor potential vanilloid 1
CaM: Calmodulin
CFA: Complete Freund's adjuvant
*AnxA1*^*−*^^*/*^^*−*^: *AnxA1* Conditional knockout
[Ca^2^^+^]i: Intracellular calcium concentration
PLCβ: Phospholipase C beta
pPLCβ: Phosphorylated PLCβ
PI3K: Phosphoinositide 3-kinase
PIP3: Phosphatidylinositol 3, 4, 5-triphosphate
DAG: Diacylglycerol
IP3: Inositol-1, 4, 5-triphosphate
ARD: Ankyrin repeat domain
