# Supplementary material for: The mechanism of Annexin A1 to modulate TRPV1 and nociception in dorsal root ganglion neurons
Source: Cell Biosci. 2021 Aug 26;11:167. doi: 10.1186/s13578-021-00679-1 (PMC8393810; doi:10.1186/s13578-021-00679-1)
Supplement: Supplementary file 1 — Additional file 1: ANXA1 is completely deleted in the DRG sensory neurons of AnxA1-/- mice. (a) Representative images of double immunofluorescence staining on cryosections of mouse L4-6 DRGs colabeled for ANXA1 and TRPV1 in control mice (Con., upper panel) and AnxA1-/- littermates (AnxA1-/-, lower panel). Scale bar, 100 μm. (b) Statistical bar graph shows the number of TRPV1 positive and FPR2 positive cells in DRG between the AnxA1-/- mice and control littermates. (c) Representative western blots band images and the quantification of the indicated proteins (d) ANXA1 (AnxA1-/- versus control group, ****P < 0.00001, Student’s t test. n=6 in WT group, n=6 in AnxA1-/- group). [file 13578_2021_679_MOESM1_ESM.pptx]

## Slide 1
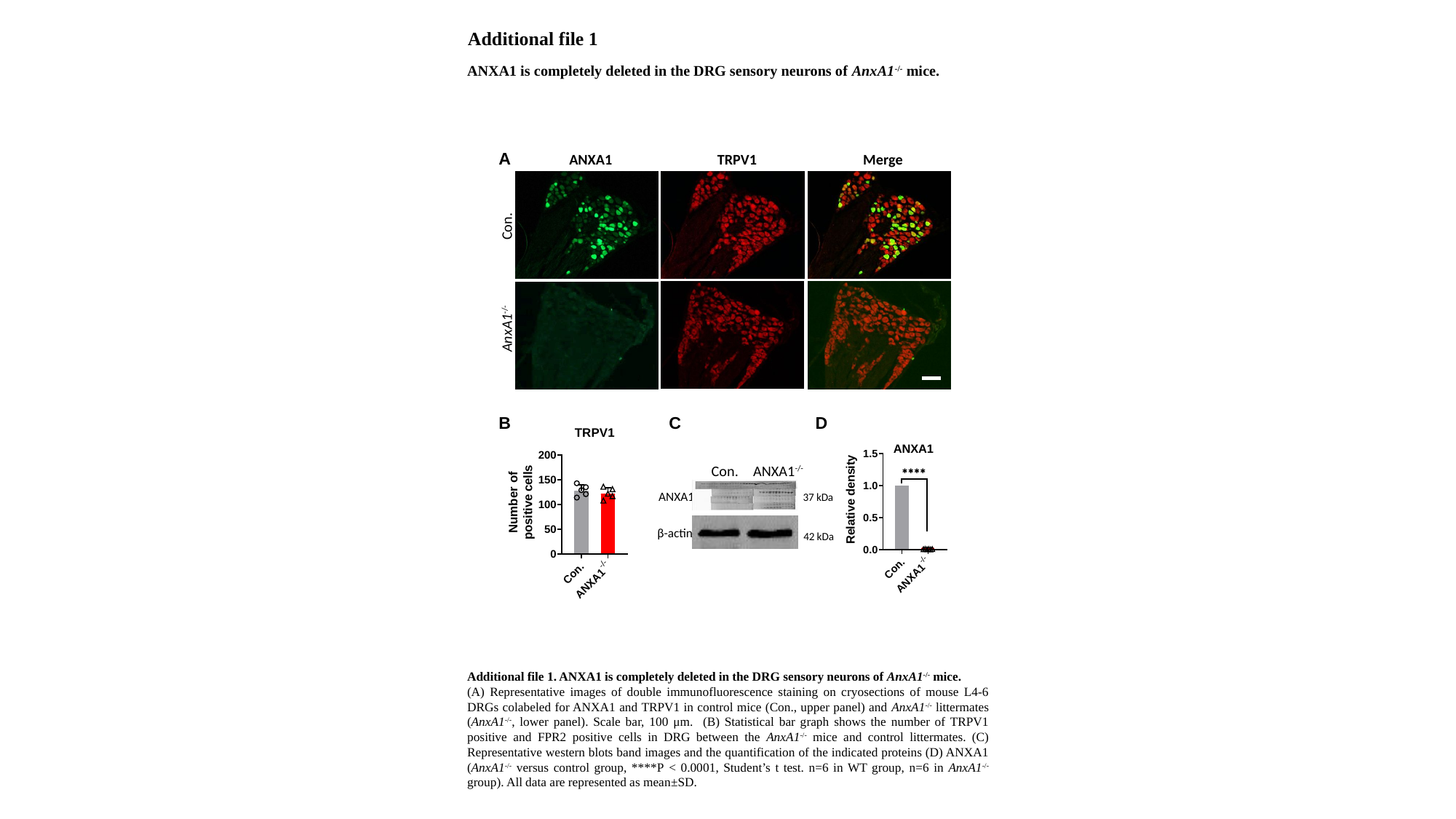

Additional file 1
ANXA1 is completely deleted in the DRG sensory neurons of AnxA1-/- mice.
A
ANXA1
TRPV1
Merge
Con.
AnxA1-/-
B
C
D
ANXA1-/-
Con.
ANXA1
37 kDa
β-actin
42 kDa
Additional file 1. ANXA1 is completely deleted in the DRG sensory neurons of AnxA1-/- mice.
(A) Representative images of double immunofluorescence staining on cryosections of mouse L4-6 DRGs colabeled for ANXA1 and TRPV1 in control mice (Con., upper panel) and AnxA1-/- littermates (AnxA1-/-, lower panel). Scale bar, 100 μm. (B) Statistical bar graph shows the number of TRPV1 positive and FPR2 positive cells in DRG between the AnxA1-/- mice and control littermates. (C) Representative western blots band images and the quantification of the indicated proteins (D) ANXA1 (AnxA1-/- versus control group, ****P < 0.0001, Student’s t test. n=6 in WT group, n=6 in AnxA1-/- group). All data are represented as mean±SD.
