# Supplementary material for: The mechanism of Annexin A1 to modulate TRPV1 and nociception in dorsal root ganglion neurons
Source: Cell Biosci. 2021 Aug 26;11:167. doi: 10.1186/s13578-021-00679-1 (PMC8393810; doi:10.1186/s13578-021-00679-1)
Supplement: Supplementary file 2 — Additional file 2: FPR2 co-expressed with TRPA1 and TRPM8 in DRG neurons of AnxA1-/- mice. (a and c) Representative images of double immunofluorescence staining on cryosections of mouse DRG co-labeled for FPR2 and TRPA1 in AnxA1-/- mice (c). (b and d) Venn diagram corresponding to the images in left panels showing the percentages of FPR2 positive and TRPA1 positive (a) or FPR2 positive and TRPM8 positive (c) neurons in DRG sections from ANXA1-/- mice as indicated. Scale bar, 100 μm. [file 13578_2021_679_MOESM2_ESM.pptx]

## Slide 1
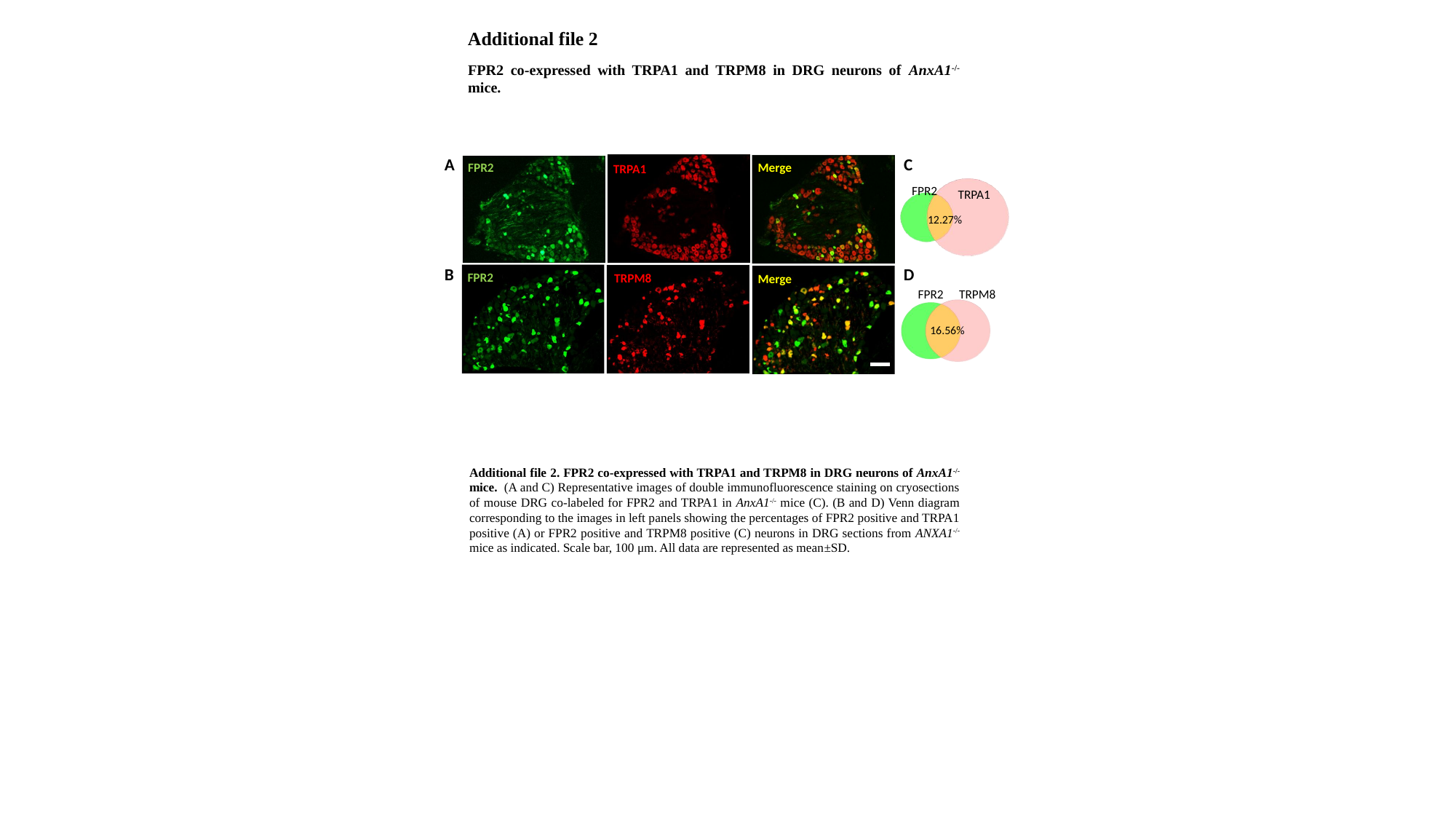

Additional file 2
FPR2 co-expressed with TRPA1 and TRPM8 in DRG neurons of AnxA1-/- mice.
A
C
FPR2
Merge
TRPA1
FPR2
TRPA1
12.27%
B
D
FPR2
TRPM8
Merge
FPR2
TRPM8
16.56%
Additional file 2. FPR2 co-expressed with TRPA1 and TRPM8 in DRG neurons of AnxA1-/- mice. (A and C) Representative images of double immunofluorescence staining on cryosections of mouse DRG co-labeled for FPR2 and TRPA1 in AnxA1-/- mice (C). (B and D) Venn diagram corresponding to the images in left panels showing the percentages of FPR2 positive and TRPA1 positive (A) or FPR2 positive and TRPM8 positive (C) neurons in DRG sections from ANXA1-/- mice as indicated. Scale bar, 100 μm. All data are represented as mean±SD.
