# Supplementary material for: The mechanism of Annexin A1 to modulate TRPV1 and nociception in dorsal root ganglion neurons
Source: Cell Biosci. 2021 Aug 26;11:167. doi: 10.1186/s13578-021-00679-1 (PMC8393810; doi:10.1186/s13578-021-00679-1)
Supplement: Supplementary file 3 — Additional file 3: Ac2-26 inhibits TRPV1 currents via FPR2 in DRG neurons of wild type mice. (a) Whole-cell current responses to applications of capsaicin (Cap.; 100 nM) + scramble, capsaicin + Ac2-26 (3.3 μM) and capsaicin + Boc2 (10 μM) + Ac2-26 (3.3 μM), respectively. (b) Statistic bar graph shows the fold change peak current in (a), scramble control was normalized to 1 for comparison. (Ac2-26 versus scramble group, ****P<0.0001; Ac2-26 versus Boc2+Ac2-26 group, ****P<0.0001, one-way ANOVA, post hoc Tukey’s multiple comparisons test, n=10 in each group). All data are represented as mean±SD. [file 13578_2021_679_MOESM3_ESM.pptx]

## Slide 1
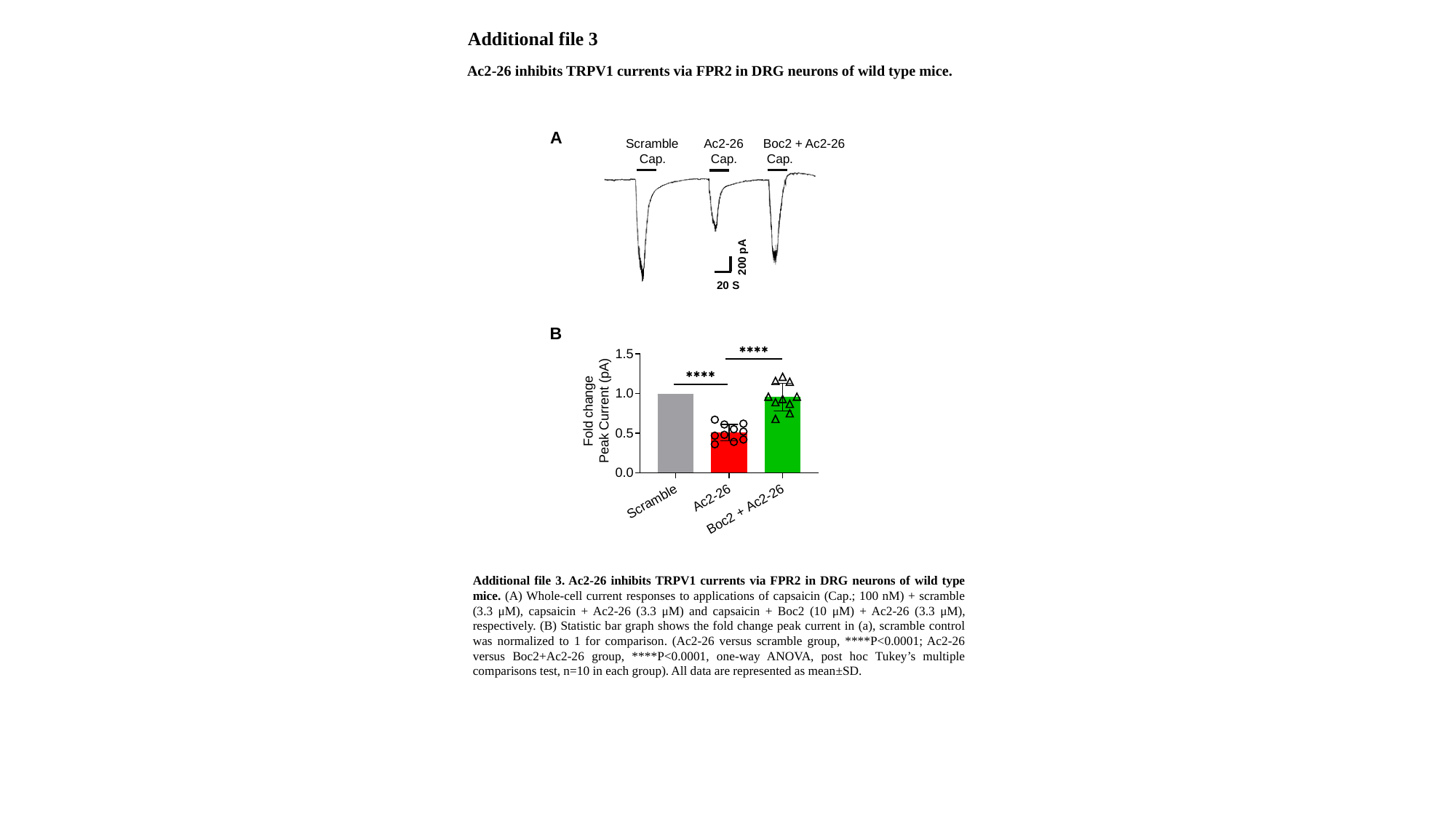

Additional file 3
Ac2-26 inhibits TRPV1 currents via FPR2 in DRG neurons of wild type mice.
A
 Scramble
 Cap.
Ac2-26
 Cap.
Boc2 + Ac2-26
 Cap.
200 pA
20 S
B
Additional file 3. Ac2-26 inhibits TRPV1 currents via FPR2 in DRG neurons of wild type mice. (A) Whole-cell current responses to applications of capsaicin (Cap.; 100 nM) + scramble (3.3 μM), capsaicin + Ac2-26 (3.3 μM) and capsaicin + Boc2 (10 μM) + Ac2-26 (3.3 μM), respectively. (B) Statistic bar graph shows the fold change peak current in (a), scramble control was normalized to 1 for comparison. (Ac2-26 versus scramble group, ****P<0.0001; Ac2-26 versus Boc2+Ac2-26 group, ****P<0.0001, one-way ANOVA, post hoc Tukey’s multiple comparisons test, n=10 in each group). All data are represented as mean±SD.
