# Supplementary material for: The mechanism of Annexin A1 to modulate TRPV1 and nociception in dorsal root ganglion neurons
Source: Cell Biosci. 2021 Aug 26;11:167. doi: 10.1186/s13578-021-00679-1 (PMC8393810; doi:10.1186/s13578-021-00679-1)
Supplement: Supplementary file 4 — Additional file 4: Ac2-26 alleviates inflammatory pain via FPR2 in AnxA1-/- mice. (a) Quantitative analysis of the licking or biting duration over 60 min after injection of 1% formalin into the hindpaw of AnxA1-/- mice (0-15 min: Ac2-26 versus scramble group, n=8, **P < 0.01; Boc2+Ac2-26 versus Ac2-26 group, n=8 *P < 0.05; 15-60 min: Ac2-26 versus scramble group, n=8, ***P < 0.001; Boc2+Ac2-26 versus Ac2-26 group, n=8 **P < 0.01; Two-way ANOVA, Sidak’s multiple comparisons test, n=8 in each group). (b) Quantitative analysis of the withdrawal latency to radiant heat in Hargreaves test (Ac2-26 versus scramble group, n=10, **P < 0.01; Boc2+Ac2-26 versus Ac2-26 group, n=10, **P < 0.01; One-way ANOVA, post hoc Tukey’s multiple comparisons test) in AnxA1-/- mice treated with after unilateral injection of CFA. [file 13578_2021_679_MOESM4_ESM.pptx]

## Slide 1
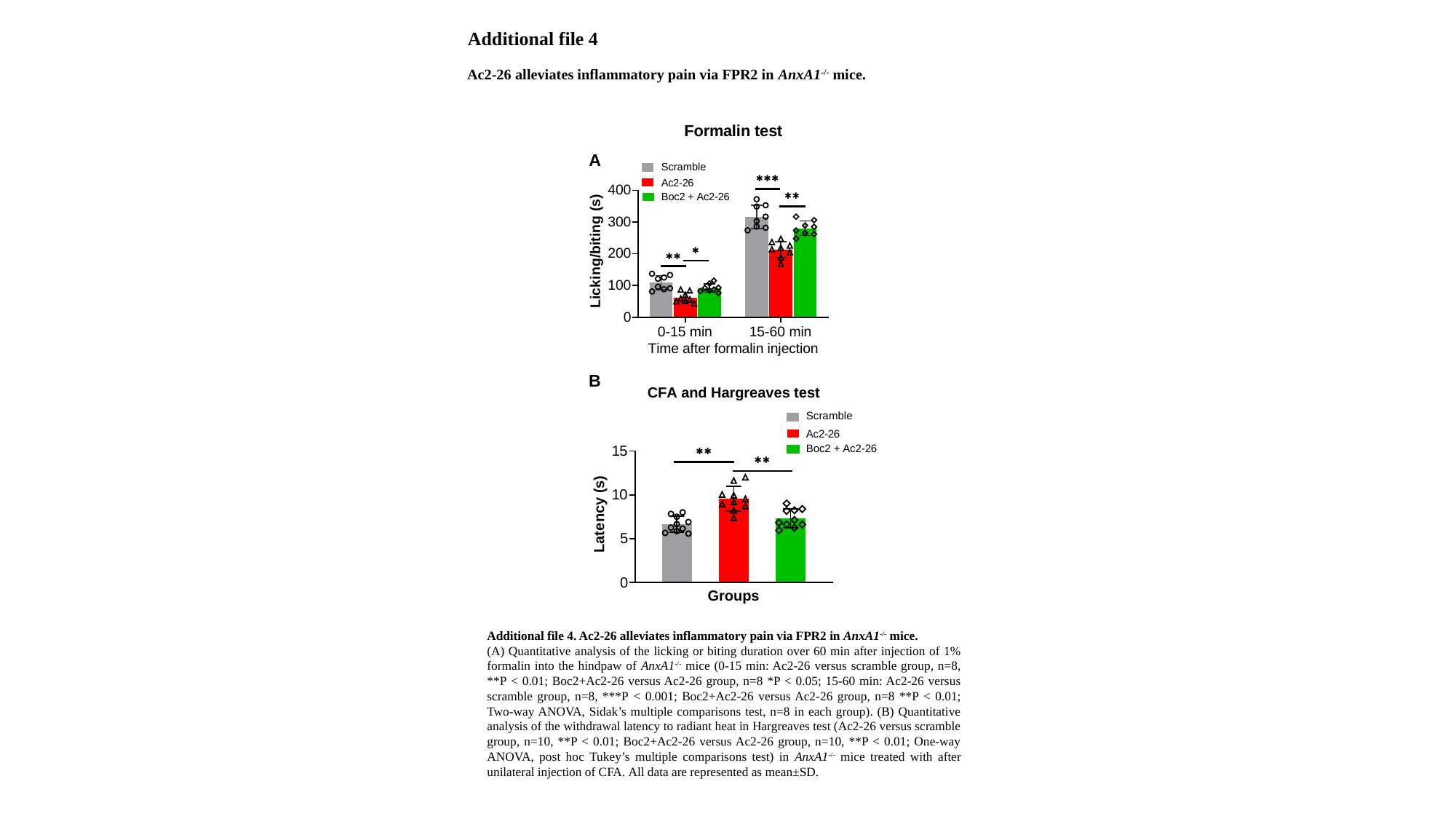

Additional file 4
Ac2-26 alleviates inflammatory pain via FPR2 in AnxA1-/- mice.
A
B
Additional file 4. Ac2-26 alleviates inflammatory pain via FPR2 in AnxA1-/- mice.
(A) Quantitative analysis of the licking or biting duration over 60 min after injection of 1% formalin into the hindpaw of AnxA1-/- mice (0-15 min: Ac2-26 versus scramble group, n=8, **P < 0.01; Boc2+Ac2-26 versus Ac2-26 group, n=8 *P < 0.05; 15-60 min: Ac2-26 versus scramble group, n=8, ***P < 0.001; Boc2+Ac2-26 versus Ac2-26 group, n=8 **P < 0.01; Two-way ANOVA, Sidak’s multiple comparisons test, n=8 in each group). (B) Quantitative analysis of the withdrawal latency to radiant heat in Hargreaves test (Ac2-26 versus scramble group, n=10, **P < 0.01; Boc2+Ac2-26 versus Ac2-26 group, n=10, **P < 0.01; One-way ANOVA, post hoc Tukey’s multiple comparisons test) in AnxA1-/- mice treated with after unilateral injection of CFA. All data are represented as mean±SD.
