# Supplementary material for: The mechanism of Annexin A1 to modulate TRPV1 and nociception in dorsal root ganglion neurons
Source: Cell Biosci. 2021 Aug 26;11:167. doi: 10.1186/s13578-021-00679-1 (PMC8393810; doi:10.1186/s13578-021-00679-1)
Supplement: Supplementary file 5 — Additional file 5: Summary graph. (Left) Genetic deletion of AnxA1 (AnxA1-/-) increases capsaicin mediated Ca2+ response and TRPV1 current in DRG neurons, and selectively enhances noxious heat or capsaicin induced pain sensation. (Right) ANXA1 mimic peptide Ac2-26 binds with FPR2, activates FPR2 coupled Gi/o signaling pathway, increases intracellular Ca2+, which binds to calmodulin (CaM) and enhances CaM-TRPV1 interaction, thus desensitizes TRPV1, finally decreases the nociceptive transmission and exerts analgesic effects. [file 13578_2021_679_MOESM5_ESM.pptx]

## Slide 1
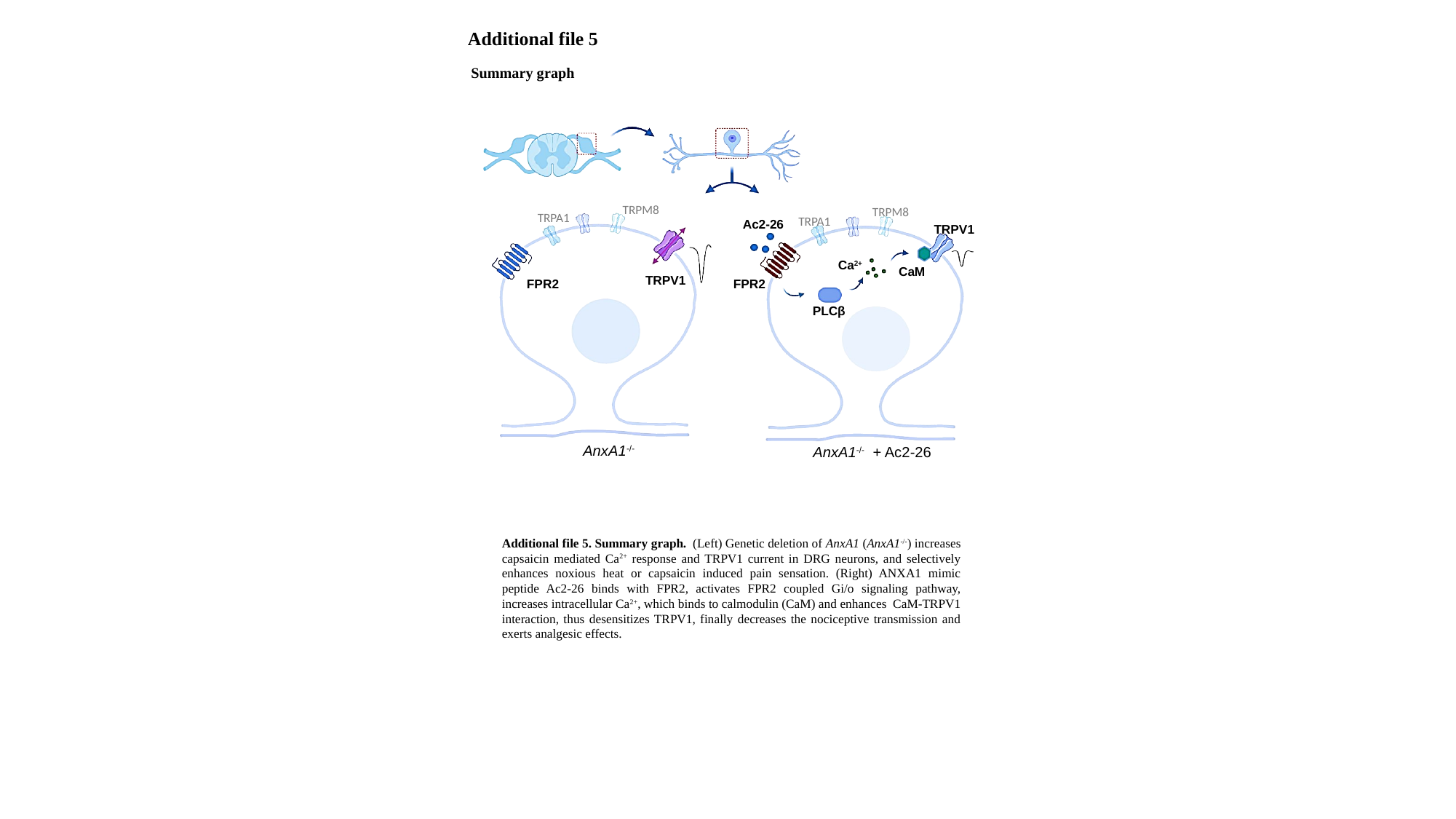

Additional file 5
Summary graph
TRPM8
TRPM8
TRPA1
TRPA1
Ac2-26
TRPV1
Ca2+
CaM
TRPV1
FPR2
FPR2
PLCβ
AnxA1-/-
AnxA1-/-
+ Ac2-26
Additional file 5. Summary graph. (Left) Genetic deletion of AnxA1 (AnxA1-/-) increases capsaicin mediated Ca2+ response and TRPV1 current in DRG neurons, and selectively enhances noxious heat or capsaicin induced pain sensation. (Right) ANXA1 mimic peptide Ac2-26 binds with FPR2, activates FPR2 coupled Gi/o signaling pathway, increases intracellular Ca2+, which binds to calmodulin (CaM) and enhances CaM-TRPV1 interaction, thus desensitizes TRPV1, finally decreases the nociceptive transmission and exerts analgesic effects.
